# Modeling a semi-optimal deceleration of a rigid body rotational motion in a resisting medium

**DOI:** 10.1038/s41598-022-22063-w

**Published:** 2022-11-07

**Authors:** F. M. El-Sabaa, T. S. Amer, A. A. Sallam, I. M. Abady

**Affiliations:** 1 Department of Mathematics, Faculty of Education, Ain Shams University, Cairo, Egypt; 2 Mathematics Department, Faculty of Science, Tanta University, Tanta, 31527 Egypt; 3 Mathematics and Computer Science Department, Faculty of Science, Suez University, Suez, 43518 Egypt

**Keywords:** Mathematics and computing, Applied mathematics, Computational science

## Abstract

This paper studies the shortest time of slowing rotation of a free dynamically asymmetric rigid body (RB), analogous to Euler’s case. This body is influenced by a rotatory moment of a tiny control torque with closer coefficients but not equal, a gyrostatic moment (GM) due to the presence of three rotors, and in the presence of a modest slowing viscous friction torque. Therefore, this problem can be regarded as a semi-optimal one. The controlling optimal decelerating law for the rotation of the body is constructed. The trajectories that are quasi-stationary are examined. The obtained new results are displayed to identify the positive impact of the GM. The dimensionless form of the regulating system of motion is obtained. The functions of kinetic energy and angular momentum besides the square module are drawn for various values of the GM’s projections on the body’s principal axes of inertia. The effect of control torques on the body's motion is investigated in a case of small perturbation, and the achieved results are compared with the unperturbed one. For the case of a lack of GM, the comparison between our results and those of the prior ones reveals a high degree of consistency, in which the deviations between them are examined. As a result, these outcomes generalized those that were acquired in previous studies. The significance of this research stems from its practical applications, particularly in the applications of gyroscopic theory to maintain the stability and determine the orientation of aircraft and undersea vehicles.

## Introduction

The problem of RB dynamics has aroused the interest of scientists to deal with it, and it is regarded as one of the important problems that have been extensively researched, due to its wide range of applications in everyday life. After the works of great scientists like Euler, Lagrange, Kovalevskaya, and others, this problem became clear due to the fact that it has been reduced to quadratures according to their cases. They pointed out the first integrable cases in the presence of some constraints on the position of the center of mass and values of the main moments of inertia^1^.

The last decades have witnessed a great interest in investigating the optimal deceleration of this problem, whether in studying integrable cases^2–4^, achieving approximate solutions using many perturbation methods^5–21^, or investigating its optimal deceleration^22–35^. In^2^, the classical Kovalevskaya top is generalized to a heavy motion of a gyrostat when the gyrostatic moment (GM) is applied and a generalization of Burn’s problem is found. Whereas, the possibility of obtaining the fourth-first integral for the RB motion through a simple procedure is investigated in^3^. The novel integrable case for the dynamics of the RB problem, which generalizes the prior cases, is discussed in^4^. This case can be described as the movment of magnetized gyrostat containing an electric charge when three classical fields are combined axially.

The approximate solutions of the RB problem are gained in many literatures e.g.^5–17^, using various perturbation approaches such as the methods of the small parameter (MSP), averaging (MA), Krylov–Bogoliubov-Mitropolsky (MKBM), and others^36,37^. In^5^, these solutions are obtained using the MSP when the motion is considered in a gravitational field and are generalized in^6,7^ to gain valid solutions at any value of the problem’s frequency when the Newtonian field and one of the third component of the GM are kept in mind. The same method is used to deal with the rotatory motion of a RB about just one fixed point when the center of mass is shifted from the dynamic axis of symmetry by a small quantity under the influence of gravitational field^8^, Newtonian field^9^, GM^9–11^, and recently by electric field^11^. The periodic analytic solutions are achieved for irrational frequency cases. The MA is used in a variety of scientific publications, e.g.^12–14^ to acquire the averaging system of the controlling one when the rotation of the body is investigated under the action of various forces and moments. The MKBM is used in^15^ and^16^ to acquire the solutions of 3D rotatory motion of asymmetric RB in a Newtonian field of force and in an electromagnetic field, respectively. Where as in^17^, the authors investigated the same motion when the body’s inertia ellipsoid is near to the body’s inertia rotation.

The governing system of a RB, in two various fields, for a case similar to Kovalevskaya is transformed to the plane motion in^18^ and^19^, respectively. Therefore, the author gained the periodic solutions of the reduced systems. Meanwhile, the impact of the GM on the RB motion is investigated in^20^ when Kovalevskaya’s conditions are applied. Two distinct methodologies are used in^21^ to examine the periodic solutions for the Hamiltonian function that governs the sextic galactic potential function. Some classes of various periodic orbits are presented through some numerical examples.

## Introduction

It is known that the nonlinear differential equations are thought to be a useful indicator of regulating feedback devices that impact the instantaneous state of the producing system's control signal. When these systems experience an impulsive disruption, we observe that these devices immediately restore the system to its equilibrium condition. These controllers are commonly employed in aircraft, spaceships, submarines, and a variety of other control systems. The progress of study in dynamics and control of bodies’ motion around a stationary point is going down the path of accounting for the reality that these bodies aren't completely rigid, but they're very close to being perfect models. Due to the rising precision demands of space exploration, gyroscopes, and other technologies, It's vital to look into the consequences of numerous deviations from perfection, in which the impact of defects can be discovered using the perturbation approaches^36^ in nonlinear mechanics.

It's worth noting that the rotation of quasi-rigid bodies without certain effective moments has never been controlled from the standpoint of impact forces at any given instant. In^22,23^, the authors used control steps and allowed themselves to use approximate approaches without taking into account the faults that resulted. Whereas in^24^, the author investigated the free movements of a symmetric body from the viewpoint of time-optimal deceleration. They supposed that the body has a globular chamber filled with liquid of high viscosity with a slow moment due to viscous frictional resistance. Whenever this model generalizes the prior works in^22,23^. Moreover, the works in^25^ and^26^ are extended to obtain optimal slowing of a confined body with torque and viscous friction that retard the body, respectively.

A minimum time for the movement of a RB connecting with a viscous elastic element as a central mass that is linked to a position on the axis of symmetry as a damper is studied in^27^. An optimal control technique is implemented, for the slowing motion of a RB rotation in which the associated time and pathways phases are estimated. However, in^28^, a semi-optimal control law is established to decelerate the asymmetric RB rotation under the influence of a tiny control torque in addition to the generalization of the examined work in^25^. The approximate solutions of a semi-optimal deceleration of a symmetric RB rotation are studied in^29^ and^30^, where the body is assumed to have a moving point mass at a fixed location on its dynamic symmetry axis. Because of the medium’s resistance, the authors assumed that the body is influenced by a restraining moment.

Controlling the bodies’ motion about a given fixed point necessitates that we take into account the fact that these bodies aren't perfectly rigid, but they're close to being perfect models. The influence of different deviations from perfection has become increasingly important in space exploration, gyroscopes, and other domains due to increasing accuracy conditions. The passive motions of stiff solid objects in a resistance medium are examined in^31–35^. The crucial fundamental issue of controlling the rotation of semi-solid bodies (the body has a hollow filled with viscous fluid, whose impact is measured by the internal torque which is generated by the viscosity of the fluid) utilizing focused torque has attracted scant attention.

The problem of damped rotatory free motion of a RB is studied in^38^. It is supposed that the body has a spherical chamber filled with a highly viscous liquid, as well as a movable mass point that is attached to the body by an elastic linkage. The law of optimal control is thus determined. In^39^, the author examined how to decelerate the rotational motion of a free RB experiencing a tiny retarding force caused by a linearly resistive medium. The mass of the body is considered to be located on the body’s symmetry dynamic axis. It is investigated in^40^ how to decelerate the rotatory motion of a symmetric RB rotation over time in a manner that is quasi-optimal. The body is supposed to have a point mass attached via a potent damper. In addition to a minor control torque with an ellipsoidal domain, the medium exerts a little linear resistive torque on the body which is related to its angular momentum. Using the MA, an asymptotic solution based on an approximate synthesis of control is presented, and a numerical investigation has been carried out. Utilizing dimensionless variables, the time-optimal slowing of asymmetric RB is investigated in^41^. For various values of the system’s parameters, a hodograph vector of the angular momentum is represented in a space. It is determined that specific ratios between the problem’s variables are required for the body’s optimal deceleration.

One of the best ways of controlling dynamical systems is the theory of Lyapunov^42^. The following is a list of the primary benefits of the strategy. This theory is realistically required when working with uncertain systems, especially nonlinear ones with time-varying parameters, and it suggests methods that are insightful and practical. However, when applied to parameters with slow time variations or continuous unknown parameters, the theory is conservative. Therefore, we propose that the total energy of a system is frequently represented by the Lyapunov function, in which its derivative gradually decreases and brings the system to equilibrium.

**Figure 1:**
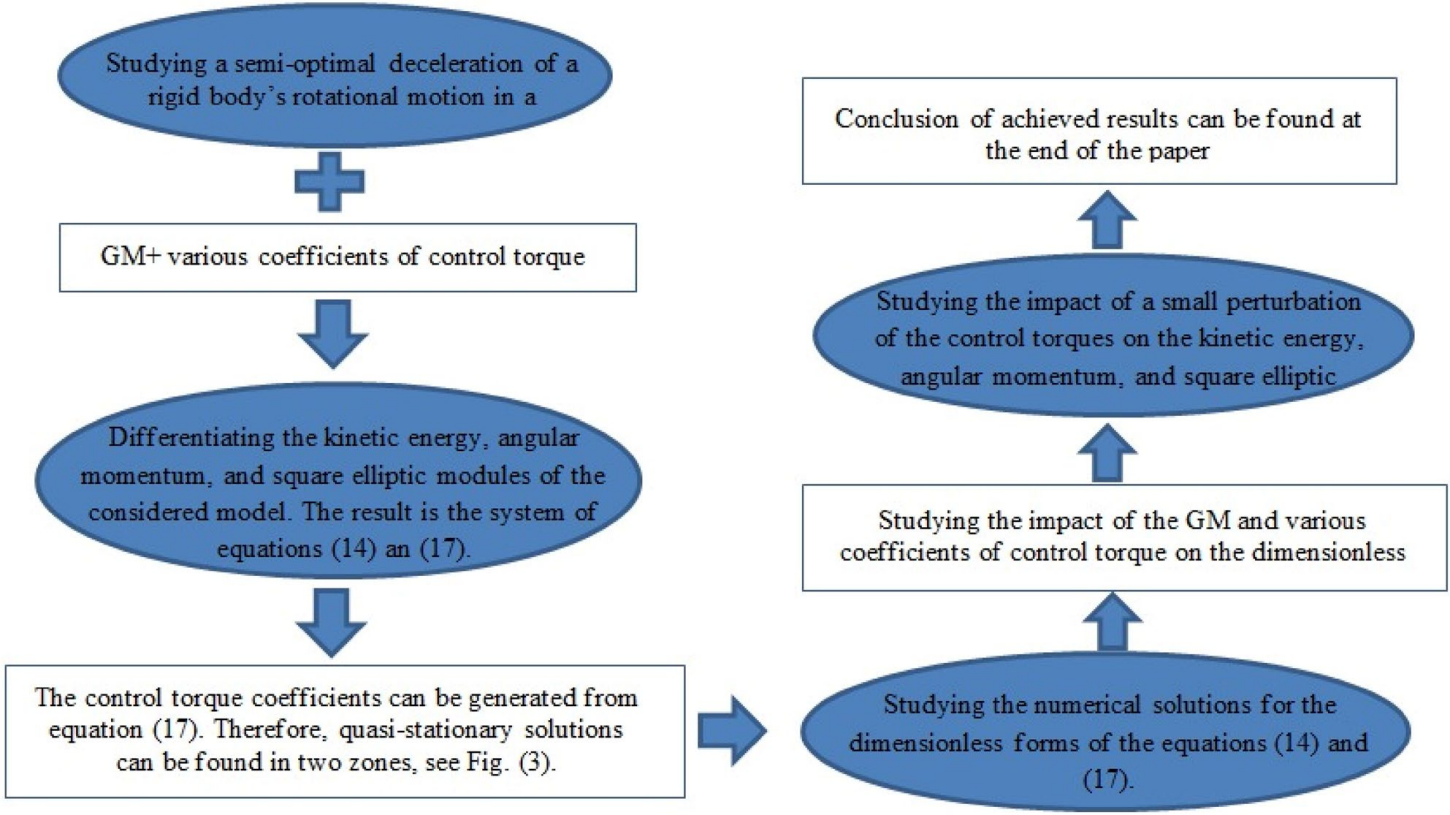
Depicts the controlled system's description.

This paper addresses the quasi-optimality deceleration for the rotatory motion of an asymmetric RB in a resistive medium under the impactness of the GM due to the presence of three rotors on the body’s principal axes, besides the activity of a bounded control torque with closer coefficients. The appropriate control law for the body’s rotation is established. The quasi-stationary trajectories are investigated. The stabilization of the RB with internal degrees of freedom is investigated. The slowdown rotations a nearly spherical RB influenced by a generated torque from the medium's linear resistance are examined. The regulating system of motion is realized in its dimensionless form. For various values of the GM components, the kinetic energy and the angular momentum functions, as well as the square module, are depicted. The terms of “optimal” and “semi-optimal” slowing are different when different values of the controlled torque dimension are selected. The effect of a tiny perturbation on the body’s motion is explored, and the results are compared with the unperturbed case. The acquired new results are graphed to determine the favorable impact of the acted GM on the body’s motion. In the absence of the gyrostat moment’s effect, we observed that our results are consistent with those of previous studies^28^, in which the discrepancies between them are evaluated. Therefore, the acquired results were more general than those obtained in earlier studies. The importance of this study arises from its practical applications, particularly in the use of gyroscopic theory to maintain stability and determine the position of aeroplanes and underwater vehicles. The sketch diagram of the considered problem is presented in Fig. 1.

## Problem’s formulation

**Figure 2:**
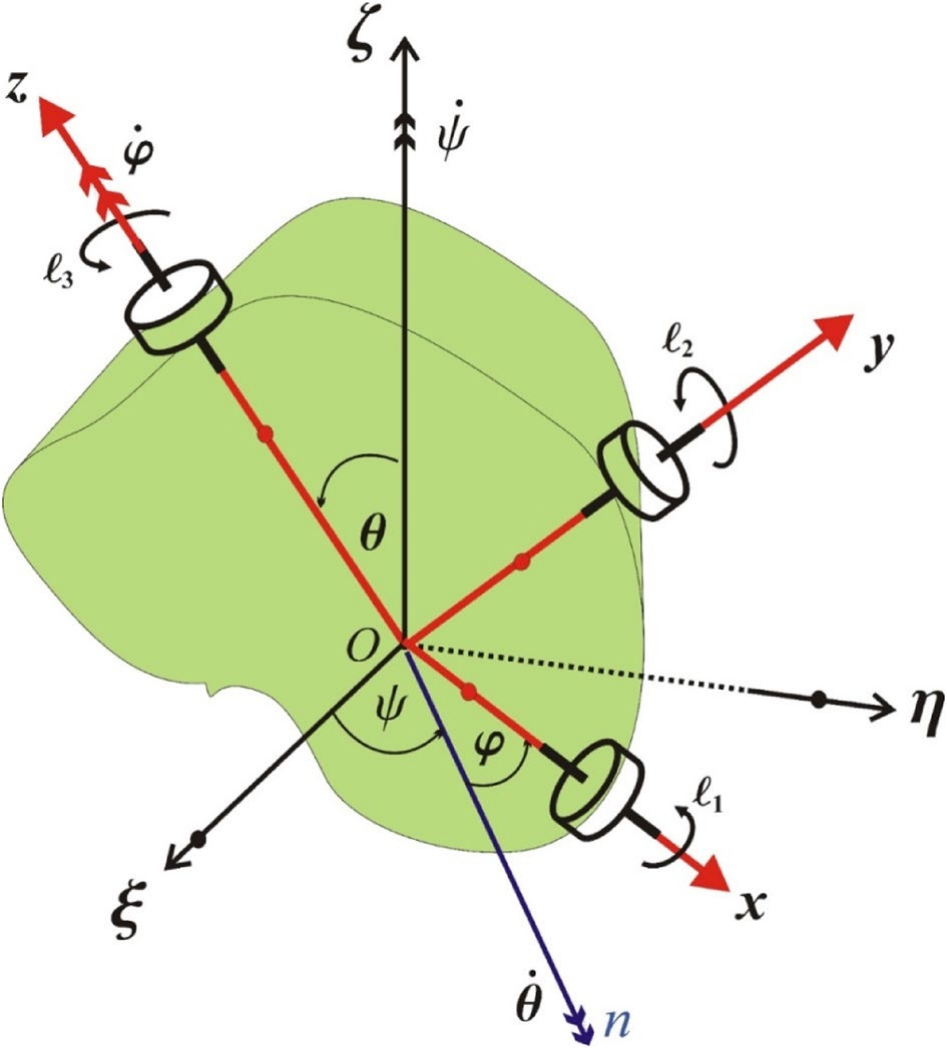
Portrays the motion of the body.

In this section, we proceed to a full illustration of the investigated problem. Therefore, we look at the rotary motion of an asymmetric RB of mass $m$ around a fixed point, say $O$. This point is considered to be the origin of two frames; the first one is fixed $\xi \eta \zeta$ and the second $xyz$ is stationary in the body and rotates with it, see Fig. 2. Three rotors are acted on the body to produce the GM $\underline{\ell }$ whose projections $(\ell_{1} ,\ell_{2} ,\ell_{3} )$ are oriented along the body’s principal axes $(Ox,Oy,Oz)$, in addition to a tiny control torque. Euler’s angles are represented by $\psi$ (the precession’s angle),$\theta$(the nutation’s angle), and $\varphi$(the proper rotation’s angle). The line $On$ is the intersection of the two planes $Oxy$ and $OXY$, in which it is referred to as a line of nodes.

We consider a RB that is dynamically asymmetric and has moments of inertia that fulfill the constraints $A > B > C$. Referring to the considered approach in^23^, the regulating equations of the rotational motion of the body according to the Euler’s case can be represented as follows^1,23,33^

1 $$\underline{{\dot{M}}} + \underline{\omega } \times \underline{M} = \underline{M}^{u} + \underline{M}^{r} ,$$

where $\underline{\omega } = {(}p{, }q{, }r{)}$ is the body’s angular velocity, whose projections on the axes $Ox,Oy$ and $Oz$ are $p,q$ and $r$, respectively, $\underline{M} = \underline{G} + \underline{\ell }$ denotes a body’s entire angular momentum, in which $\underline{G} = J\underline{\omega }$, $J = {\text{diag (}}A{,}\;B{,}\;C{)}$ is the body’s inertia tensor, and dots indicate the derivatives regarding time $t$. Then, one writes

2 $$M = \left| {\underline{M} } \right| = [(G_{1} + \ell_{1} )^{2} + (G_{2} + \ell_{2} )^{2} + (G_{3} + \ell_{3} )^{2} ]^{{{1 \mathord{\left/ {\vphantom {1 2}} \right. \kern-\nulldelimiterspace} 2}}} .$$

In (1), $\underline{M}^{u}$ represents the control torque’s vector and $\underline{M}^{r}$ denotes the torque of dissipation that is assumed proportionally with the angular momentum; i.e.

3 $$\underline{M}^{r} = - \lambda ^{\prime}\,\underline{M},$$

where $\lambda^{\prime}$ refers to a constant coefficient that depends on the medium’s properties. The medium's resistance is expected to be minimal, with a tiny order of magnitude, i.e., $\lambda^{\prime} = \varepsilon \lambda$ where $\varepsilon < < 1$ is very tiny. In this case, $\varepsilon {\kern 1pt} \lambda \,M_{j} \,(j = 1,2,3)$ is the momentum’s projections on the major principal axes of body inertia^33^.

It is supposed that the control torque $\underline{M}^{u}$ has a tiny magnitude and can be represented in terms of order of $\varepsilon$, in which its components are expressed by the products of $b_{j}$ which is a constant with a torque dimension, by the tiny parameter $\varepsilon$ and by the nondimensional control functions $u_{j}$ that can be estimated. Therefore, we can write

4 $$M_{j}^{u} = \varepsilon \,b_{j} \,u_{j} .$$

The outcomes of $\varepsilon \,b_{j} \,(j = 1,{\kern 1pt} 2,{\kern 1pt} 3)$ describe the control system’s efficiency in relation to the corresponding body’s principal axes. Therefore, the governing equations of motion (1) can be rewritten as follows

5 $$\begin{gathered} \dot{M}_{1} + M_{3} q - M_{2} r = \varepsilon b{}_{1}u_{1} - \varepsilon \lambda \,M_{1} , \hfill \\ \dot{M}_{2} + M_{1} r - M_{3} p = \varepsilon b{}_{2}u_{2} - \varepsilon \lambda \,M_{2} , \hfill \\ \dot{M}_{3} + M_{2} p - M_{1} q = \varepsilon b{}_{3}u_{3} - \varepsilon \lambda \,M_{3} . \hfill \\ \end{gathered}$$

In view of the equations of system (5), it is necessary to achieve the best possible controls $u_{j} = u(t,p,q,r)$ which fulfil the following constraint

6 $$u_{1}^{2} + u_{2}^{2} + u_{3}^{2} \le 1,$$

and (based on the above system) from a given initial state $\underline{\omega } \,(t_{0} ) = \underline{\omega }^{0}$ into the case of a rest state $\underline{\omega } \,(T) = \underline{0}$ in a minimum amount of time.

In the case $b_{j} = b > 0\,(j = 1,\,2,\,3)$, in which $b$ is a time-dependent function, then the best control can thus be expressed as $u_{j} = - {{M_{j} } \mathord{\left/ {\vphantom {{M_{j} } M}} \right. \kern-\nulldelimiterspace} M}$. Here, $u_{j}$ represent the projections of the vector $\underline{u}$ on the body’s principal axes^23,43^. This control can be characterized as a semi-optimal control if $b_{j}$ are near to each other as in^23,44^.

For applications, it’s interesting to look into the rigid bodies’ motions with a basic control described as follows^23,44^

7 $$M_{j}^{u} = \varepsilon \,b_{j} u_{j} ,\quad u_{j} = - {{M_{j} } \mathord{\left/ {\vphantom {{M_{j} } M}} \right. \kern-\nulldelimiterspace} M};\quad j = 1,2,3.$$

Incorporating (7) into (5) to produce a complete controlling system of motion related to projections on the inertia principle axes; thus, the kinematic relationships are not written out here.

### The problem’s proposed solution

In this section, we investigate the solution of the semi-optimal slowing problem. To achieve this purpose, bearing in mind Eqs. (7) and multiplying equations of system (5) by $M_{1} ,\,M_{2}$, and $M_{3}$, respectively. Adding the result, to yield the dot product $(\underline{{\dot{M}}} \cdot \underline{M} )$. Taking into consideration the derivative’s property of the dot squared product $\underline{{\dot{M}}} \cdot \underline{M} = {{d(M^{2} )} \mathord{\left/ {\vphantom {{d(M^{2} )} {(2dt) = }}} \right. \kern-\nulldelimiterspace} {(2dt) = }}M\,\dot{M}$, then multiplying the result by $(1/M)$ to get the following scalar equation

8 $$\dot{M} = - \varepsilon \lambda M - \frac{\varepsilon }{{M^{2} }}(b_{1} M_{1}^{2} + b_{2} M_{2}^{2} + b_{3} M_{3}^{2} ).$$

Based on the gyrostat’s kinetic energy $H$^2^ and its first derivative^25^, we can express them in the following forms

9 $$\begin{aligned} H & = \frac{1}{2}[Ap^{2} + Bq^{2} + Cr^{2} + 2(\ell_{1} p + \ell_{2} q + \ell_{3} r)], \\ \dot{H} & = - \varepsilon \,H - \frac{\varepsilon }{M}\left( {\frac{{b_{1} }}{A}M_{1}^{2} + \frac{{b_{2} }}{B}M_{2}^{2} + \frac{{b_{3} }}{C}M_{3}^{2} } \right). \\ \end{aligned}$$

If $\varepsilon = 0$, then we have the unperturbed case and therefore, the body’s rotation corresponds to the case of Euler–Poinsot. Moreover, the Euler’s angles $\theta ,\,\phi$, and $\psi$ are time-dependent, while the parameters $M$ and $H$ are constants. On the other hand, for the perturbed case ($\varepsilon \ne 0$) Euler’s angles are given in terms of fast variables, whereas $M$ and $H$ are expressed in terms of slow variables. As explained earlier, we investigate the problem’s statement of slowing rotation where there are no angle variables that can be determined by integrating the dynamic and kinematic equations simultaneously.

Now, we analyze the motion described by the constraint $2AH \ge M^{2} \ge 2CH$, that corresponds to the vector of angular momentum’s paths which surrounds the greatest axes of the inertia moment $Oz$. The elliptic function module $k$^31^ can be determined as follows:

Solving Eq. (2) and the first one of Eq. (9) together, to get

10 $$\begin{aligned} ([p + (\ell_{1} /A)]^{2}) & = \frac{B(B - C)}{{A(A - C)}}\{ f^{2} - [q + (\ell_{2} /B)]^{2} \} , \\ ([r + (\ell_{3} /C)]^{2}) & = \frac{B(B - A)}{{C(C - A)}}\{ g^{2} - [q + (\ell_{2} /B)]^{2} \} , \\ \end{aligned}$$

where

$$\begin{gathered} f^{2} = \frac{{M^{2} - 2CH - L_{1} }}{B(B - C)},\quad \quad \quad \quad g^{2} = \frac{{M^{2} - 2AH - L_{2} }}{B(B - A)}, \hfill \\ L_{1} = ({C \mathord{\left/ {\vphantom {C A}} \right. \kern-\nulldelimiterspace} A})\ell_{1}^{2} + ({C \mathord{\left/ {\vphantom {C B}} \right. \kern-\nulldelimiterspace} B})\ell_{2}^{2} + \ell_{3}^{2} ,\quad \quad L_{2} = \,\ell_{1}^{2} + ({A \mathord{\left/ {\vphantom {A B}} \right. \kern-\nulldelimiterspace} B})\ell_{2}^{2} + ({A \mathord{\left/ {\vphantom {A B}} \right. \kern-\nulldelimiterspace} B})\ell_{3}^{2} . \hfill \\ \end{gathered}$$

Referring to the above, the formula of the square module function $k$ is as follows

11 $$k^{2} = \frac{{f^{2} }}{{g^{2} }} = \frac{{(B - C)(2AH + L_{1} - M^{2} )}}{{(A - B)(M^{2} - 2CH - L_{2} )}},\quad \quad \quad \quad 0 \le k^{2} \le 1.$$

Therefore, the value of $k$ that describes the elliptic modulus remains constant for the unperturbed case, and it has a one-of-a kind relationship with angular momentum $M$ and kinetic energy $H$.

From^45^ we can get

12 $$\begin{gathered} \left( {p + \frac{{\ell_{1} }}{A}} \right)^{2} = p_{{_{{^{1} }} }}^{2} = \frac{{M^{2} - 2CH}}{A(A - C)}\frac{E(k)}{{K(k)}}, \hfill \\ \left( {q + \frac{{\ell_{2} }}{B}} \right)^{2} = q_{{_{{^{1} }} }}^{2} = \frac{{2AH - M^{2} }}{B(A - B)}(1 - \frac{E(k)}{{K(k)}}), \hfill \\ \left( {r + \frac{{\ell_{3} }}{C}} \right)^{2} = r_{{_{{^{1} }} }}^{2} = \frac{2AH}{{C(A - B)k^{2} }}\left( {\frac{E(k)}{{K(k)}} + k^{2} - 1} \right). \hfill \\ \end{gathered}$$

Making use of Eq. (2) and the first one of Eq. (9) to obtain

13 $$\frac{{M^{2} }}{2H} = \frac{{\beta_{1} }}{{\beta_{2} }},$$

where

$$\begin{aligned} \beta_{1} & = A(B - C) + (A - C)\,C{\kern 1pt} k^{2} , \\ \beta_{2} & = (B - C) + (A - B)\,k^{2} . \\ \end{aligned}$$

Therefore, one can write the derivatives of $M$ and $H$ as follows

14 $$\begin{aligned} \dot{M} & = - \varepsilon \lambda M - \frac{\varepsilon }{{M^{2} }}(b_{1} A^{2} p_{{_{{^{1} }} }}^{2} + b_{2} B^{2} q_{{_{{^{1} }} }}^{2} + b_{2} C^{2} r_{{_{{^{1} }} }}^{2} ), \\ \dot{H} & = - \varepsilon \,H - \frac{\varepsilon }{M}(b_{1} Ap_{{_{{^{1} }} }}^{2} + b_{2} Bq_{{_{{^{1} }} }}^{2} + b_{3} Cr_{{_{{^{1} }} }}^{2} ). \\ \end{aligned}$$

We insert $(p_{1} ,\,q_{1} ,\,r_{1} ) = \left( {p + \frac{{\ell_{1} }}{A},q + \frac{{\ell_{2} }}{B},r + \frac{{\ell_{3} }}{C}} \right)$, where the projections $p,q,$ and $r$ of $\underline{\omega }$ from the unperturbed motion of Euler–Poinsot^45^, into the right sides of (14) and then average across the duration of this motion to obtain the averaged first approximation system. The same abbreviation for the slow variables $M$ and $H$ can be used. Consequently, for $\tau = \varepsilon \,t\, \in [0,T]$, we can get

15 $$\begin{aligned} \frac{dH}{{d\tau }} & = - 2\lambda H - \frac{M}{\alpha }\left\{ {b_{1} (B - C)\frac{E(k)}{{K(k)}} + b_{2} (A - C)\left[ {1 - \frac{E(k)}{{K(k)}}} \right] + b_{3} (A - B)\left[ {\frac{E(k)}{{K(k)}} - K^{2} - 1} \right]} \right\}, \\ \frac{dM}{{d\tau }} & = - \lambda M - \frac{1}{\alpha }\{ b_{1} A(B - C)\frac{E(k)}{{K(k)}} + b_{2} B(A - C)W(k) + b_{3} C(A - B)[K^{2} - W(k)]\} , \\ \end{aligned}$$

where

$$\alpha = A(B - C) + C(A - B)k^{2} ,\;W = 1 - \frac{E(k)}{{K(k)}},$$

where $K(k)$ and $E(k)$ represent the first and second kinds of full elliptic integrals^46^, respectively. According to the first equation in (15), the body's kinetic energy $H$ progresses under the effect of drag’s medium, control torque, and the GM $\underline{\ell }$. The expression’s value of the brackets located in the right side of the first equation of (15) is non-negative for $A > B > C$ owing to the inequalities^46^

16 $$(1 - k^{2} )K(k) \le E(k) \le K(k).$$

Since $H > 0$, we find $\frac{dH}{{d\tau }} < 0$. Then $H$ represents a Lyapunov function^42^ for any $k^{2} \in [0,1][0,1]$; $H$ is always decreasing. We can also demonstrate the decrease of the angular momentum.

Differentiating the module formula (12) and taking into account the derivatives in (15), to yield the following differential equation

17 $$\begin{aligned} \frac{{dk^{2} }}{d\tau } & = (A - B)^{ - 2} (M^{2} - 2CH - L_{2} )^{ - 2} \{ (A - B)(B - C)\{ - 2\lambda [(M^{2} - 2CH - L_{2} ) \\&\quad \times \;(2AH - M^{2} ) - (2AH + L_{1} - M^{2} )(M^{2} - 2CH)] - \frac{{2M^{2} }}{{\alpha_{1} }}(A - C)[(A - B) \\&\quad \left. {\left. {\left. { \times \;(M^{2} - 2CH - L_{2} )(b_{2} W + b_{3} (k^{2} - W) - (B - C)(2AH + L_{1} - M^{2} )( {b_{1} \frac{E}{K} + b_{2} W} )} \right]} \right\}} \right\}. \\ \end{aligned}$$

Let $L_{2} = \frac{{M^{2} - 2CH}}{{2AH - M^{2} }}L_{1}$, then Eq. (17) can be rewritten as follows

18 $$\frac{{dk^{2} }}{d\tau } = \frac{2M(A - C)}{{[M^{2} (A - C) - AL_{2} + CL_{1} ]}}\left[ {b_{1} k^{2} \frac{E}{K} + b_{2} W(k^{2} - 1) + b_{3} (W - k^{2} )} \right].$$

## Examination of quasi-steady kinematics

This section discusses a differential singularity in the present work as it relates to other works; the impact of control torque, and dissipating forces on the evolution of $k$, the time histories of the deceleration as a function of the magnitude of $b$, and the regions of semi-stationary solutions are determined.

It must be noted that there is an essential singularity in^28^ when $M \to 0$, in which the authors examined the problem in the absence of gyrostatic moment. Therefore, the terms $L_{1}$ and $L_{2}$ don’t appear in the same work, and coqusequently, the singularity has been arisen. In the present work, the singularity does not exist at all, since it is impossible $M^{2} = {{(AL_{2} - CL_{1} )} \mathord{\left/ {\vphantom {{(AL_{2} - CL_{1} )} {(A - C}}} \right. \kern-\nulldelimiterspace} {(A - C}})$. The reasone is due to the presence of the GM, that appears in the terms $L_{1}$ and $L_{2}$, which reinforces the importance of its influence on the body’s dynamical behavior. For the case $k^{2} = 1$ which is equivalent to $2BH = M^{2}$, we can find that it is correlated to the separatrix for the Euler–Poinsot motion. Equation (14) characterizes the averaging motion of the angular momentum vector’s terminal on a sphere with a radius $M$.

The control torque as well as the torque generated by the forces of dissipation have an impact on the evolution of $k^{2}$. The stationary fixed points $k_{*}^{2}$ for the differential Eq. (14); as well as $k_{*}^{2} = 0$ and $k_{*}^{2} = 1$, have the form

19 $$k_{*}^{2} = (b_{2} - b_{3} )W(k_{*} )[(b_{1} - b_{2} )\frac{{E(k_{*} )}}{{K(k_{*} )}} + (b_{2} - b_{3} )]^{ - 1} .$$

It's important to note that, the motion of $\underline{M}$ is often constituted exclusively of the motion along the path of Euler–Poinsot and decreases in the length of $\underline{M}$ with time for the the RB semi-stationary motions that match the stationary locations $k_{*}^{2}$.

In the case of dimensionless quantities of $k_{*}^{2}$ the next notation can be introduced

20 $$\,\kappa_{1} = \frac{{b_{1} }}{{b_{3} }},\quad \kappa_{2} = \frac{{b_{2} }}{{b_{3} }},$$

and then (18) becomes

21 $$\,k_{*}^{2} = (\kappa_{1} - \kappa_{2} )W(k_{*} )\left[ {(\kappa_{1} - \kappa_{2} )\frac{{E(k_{*} )}}{{K(k_{*} )}} + (\kappa_{2} - 1)} \right]^{ - 1} .$$

This means that

22 $$\kappa_{1} = (\kappa_{2} - 1)\frac{{[W(k_{*} ) + k_{*}^{2} F(k_{*} ) - k_{*}^{2} ]}}{{k_{*}^{2} F(k_{*} )}} + \frac{{[k_{*}^{2} - W(k_{*} )]}}{{k_{*}^{2} F(k_{*} )}};\,\,\,\,\,\,\,F(k_{*} ) = \frac{{E(k_{*} )}}{{K(k_{*} )}}.$$

Based on the previous formula (22), one can conclude that it is a linear function when the requirements $\kappa_{1} > 0$ and $\kappa_{2} > 0$ are satisfied. If the second one is met, then we can get

23 $$\,\kappa_{2} > \frac{{W(k_{*} ) - k_{*}^{2} }}{{W(k_{*} ) + k_{*}^{2} F(k_{*} ) - k_{*}^{2} }}.$$

**Figure 3:**
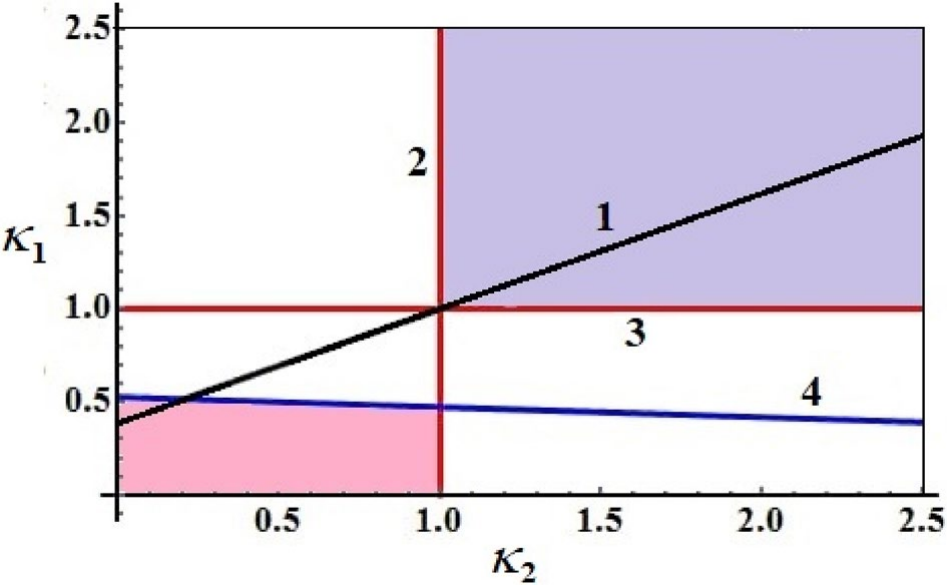
Sketches the areas of quasi-stationary solutions.

It is clear that, the right hand side of the previous inequality (23) will be positive for any value of $k_{*}^{2}$, if the inequalities (16) are holded. As a result, for all $\kappa_{2}$ fulfilling the inquality (23), the required condition must be satisfied too. The essential conditions for the presence of semi-stationary solutions for $\kappa_{1}$ and $\kappa_{2}$ can be obtained through satisfying the inquality $0 < k_{*}^{2} < 1$ in the left hand side of Eq. (21). There are two zones where in quasi-stationary solutions can be found, see Fig. 3.

It is noted that the establishment of these zones can be achieved as follows; the boundary lines are line 2 which matches to $\kappa_{2} = 1$, line 3 that matches to $\kappa_{1} = 1$, and line 4 which is specified by the following equation

24 $$\,\kappa_{1} = \frac{1}{{F(k_{*} )}}\left[ {1 - \kappa_{2} \left( {1 - \frac{{E(k_{*} )}}{{K(k_{*} )}}} \right)} \right].$$

The inspection of Fig. 3 shows that, line 1 is drawn according to Eq. (22) at $k_{*}^{2} = 0.4$. Moreover, it has been observed that for some certain values of the dimensionless parameters for the projections $\kappa_{1}$ and $\kappa_{2}$ of the control torque, a quasi-stationary movment occurs, but not for all, in which the zones defined earlier can have linear dependence (22).

Consider the equation that governs the system’s variation of the angular momentum (15), as well as Eq. (18).

**Figure 4:**
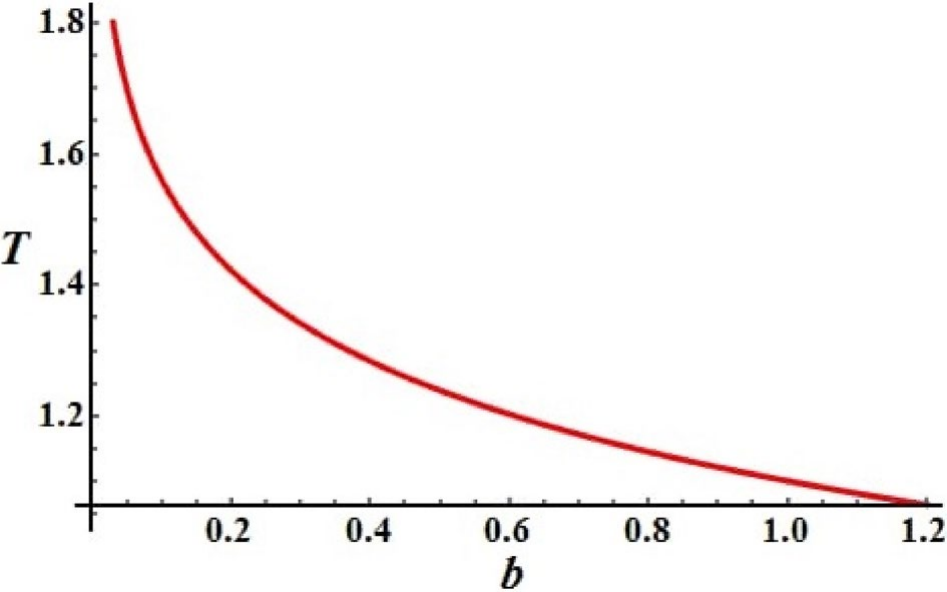
Explains the control torque’s coefficients.

We investigate the RB's deceleration time as a function of the magnitude of $$b_{j} \,(j = 1,2,3)$$ for the control torque. Figure 4 is graphed at $$A = 10\,{\text{kg}}\;{\text{m}}^{2} ,B = 8\,{\text{kg}}\;{\text{m}}^{2} ,\,C = 5\,{\text{kg}}\;{\text{m}}^{2} ,$$$$\ell_{1} = 40\;{\text{kg}}\;{\text{m}}^{2} \;{\text{s}}^{ - 1} ,\ell_{2} = 30\;{\text{kg}}\;{\text{m}}^{2} \;{\text{s}}^{ - 1} ,$$ and $$\ell_{3} = 20\;{\text{kg}}\;{\text{m}}^{2} \;{\text{s}}^{ - 1} ,$$ in addition to the intial values of the angular velocity $$p_{0} = 0.01\,{\text{rad}}\;{\text{s}}^{ - 1} ,q_{0} = 0.03\,{\text{rad}}\;{\text{s}}^{ - 1} ,$$ and $$r_{0} = 0.03\,{\text{rad}}\;{\text{s}}^{ - 1} .$$ The potted curve describes the coefficient of the control torque, it can be observed that the function has an exponential form.

The greatest value of this curve is identical to the lowest value of $b$ and a gradual decrease of the curve is observed till the end of the range of $b$. It is worthwhile to mention that the $T = T(b_{j} )$ function defined in^26^ has the same behavior to some extent.

## Numerical results

This section aims to solve the systems of Eqs. (15) and (18) numerically in accordance with the different values of gyrostatic moment and control torque. To accomplish this purpose, we are going to transform the mentioned system to its dimensionless form by using the system’s parameters like the deceleration time $T$, the intial value of angular momentum value $M_{0}$, and the control torque’s cefficient $b_{3}$. Then, we can write.

Based on The system equations in the form of dimensionless is

25 $$\begin{gathered} \tilde{t} = \frac{\tau }{T},\,\,\,\,\,\,\,\tilde{\lambda } = \lambda \,T,\,\,\,\,\,\,\,\tilde{H} = \frac{H}{{b_{3} }},\,\,\,\,\,\,\,\tilde{A} = \frac{A}{{M_{0} T}},\,\,\,\,\,\,\,\tilde{B} = \frac{B}{{M_{0} T}}\,,\,\, \hfill \\ \,\tilde{C} = \frac{C}{{M_{0} T}},\,\,\,\,\,\,\,\tilde{M} = \frac{M}{{M_{0} }},\,\,\,\,\,\,\,\,\alpha = M_{0}^{2} \,T^{2} \,\tilde{\alpha },\,\,\,\,\,\,\,\,\tilde{L}_{j} = \frac{{L{}_{j}}}{{M_{0}^{2} }}\;\;(j = 1,2).\,\,\, \hfill \\ \end{gathered}$$

Consider the following identifying number

26 $$\sigma = \frac{{b_{3} T}}{{M_{0} }},$$

which specifies the fundamental process of the RB’s deceleration under the influence of a control torque in the shortest or minimum possible period $T$.

Referring to the above dimensionless forms and definition of $\sigma$, the dimensionless form of the system of Eqs. (15) and (18) has the form

27 $$\begin{aligned} \frac{{d\tilde{H}}}{{d\tilde{t}}} & = - 2\tilde{\lambda }\tilde{H} - \frac{{\tilde{M}}}{{\tilde{\alpha }}}\left\{ {\kappa_{1} (\tilde{B} - \tilde{C})\frac{E(k)}{{K(k)}} + \kappa_{2} (\tilde{A} - \tilde{C})\left[ {1 - \frac{E(k)}{{K(k)}}} \right] + (\tilde{A} - \tilde{B})\left[ {\frac{E(k)}{{K(k)}} - k^{2} - 1} \right]} \right\}, \\ \frac{{d\tilde{M}}}{{d\tilde{t}}} & = - \tilde{\lambda }\tilde{M} - \frac{\sigma }{{\tilde{\alpha }}}\left\{ {\kappa_{1} \tilde{A}(\tilde{B} - \tilde{C})\frac{E(k)}{{K(k)}} + \kappa_{2} \tilde{B}(\tilde{A} - \tilde{C})W(k) + \tilde{C}(\tilde{A} - \tilde{B})[k^{2} - W(k)]} \right\}, \\ \frac{{dk^{2} }}{{d\tilde{t}}} & = \frac{2\sigma }{{[\tilde{M}^{2} (\tilde{A} - \tilde{C}) - \tilde{A}\tilde{L}_{2} + \tilde{C}\tilde{L}_{1} ]}}\left\{ {\left[ {\kappa_{1} k^{2} \frac{E}{K} + \kappa_{2} W(k^{2} - 1) + (W - k^{2} )} \right]} \right\}, \\ \end{aligned}$$

where

$$\tilde{\alpha } = \tilde{A}(\tilde{B} - \tilde{C}) + \tilde{C}(\tilde{A} - \tilde{B})k^{2} .$$

The integration was carried out for the beginning circumstances $k^{2} (0) = 0.99$ and $\tilde{M}(0) = 1$; in which the initial value of the kinetic energy can be calculated using the next equation

28 $$\tilde{H}(0) = \frac{{(\tilde{B} - \tilde{C})(\tilde{L}_{1} - \tilde{M}^{2} ) - k^{2} (\tilde{A} - \tilde{B})(\tilde{M}^{2} - \tilde{L}_{2} )}}{{2\sigma \tilde{\alpha }}}.$$

It must be mentioned that the variation of $k^{2}$ can be described by the third Eq. (27). Thus, the right hand side of the mentioned equation must be negative for the initial circumstance $k^{2} \approx 1$. Moreover, in the bracket, the third term is negative. Consequently, the below condition

29 $$\kappa_{1} < \frac{{\kappa_{2} (1 - k^{2} )W + k^{2} - W}}{{k^{2} F}},$$

for the control torque's dimensionless coefficients must be satisfied.

To achieve the numerical solution of the system (27), we can take into account the dimensionless form (25) beside the following data

$$\begin{aligned} A & = 35\;{\text{kg}}\;{\text{m}}^{2} ,\;\;\;B = 22\;{\text{kg}}\;{\text{m}}^{2} ,\;\;\;\;C = 16\;{\text{kg}}\;{\text{m}}^{2} ,\;\;\;\;p_{0} = 0.01\;{\text{rad}}\;{\text{s}}^{ - 1} , \\ q_{0} & = 0.02\;{\text{rad}}\;{\text{s}}^{ - 1} ,\;\;\;\;r_{0} = 0.03\;{\text{rad}}\;{\text{s}}^{ - 1} ,\;\;\;\;\lambda = 0.2. \\ \end{aligned}$$

In addition to the selected values of the GM $\underline{\ell }$ components, the values of the dimensionless coefficients of the torques $(\kappa_{1} ,\kappa_{2} )$, and the identifying number $\sigma$, where the RB’s deceleration is quasi-optimal. Deceleration can occur in a variety of ways.

**Figure 5:**
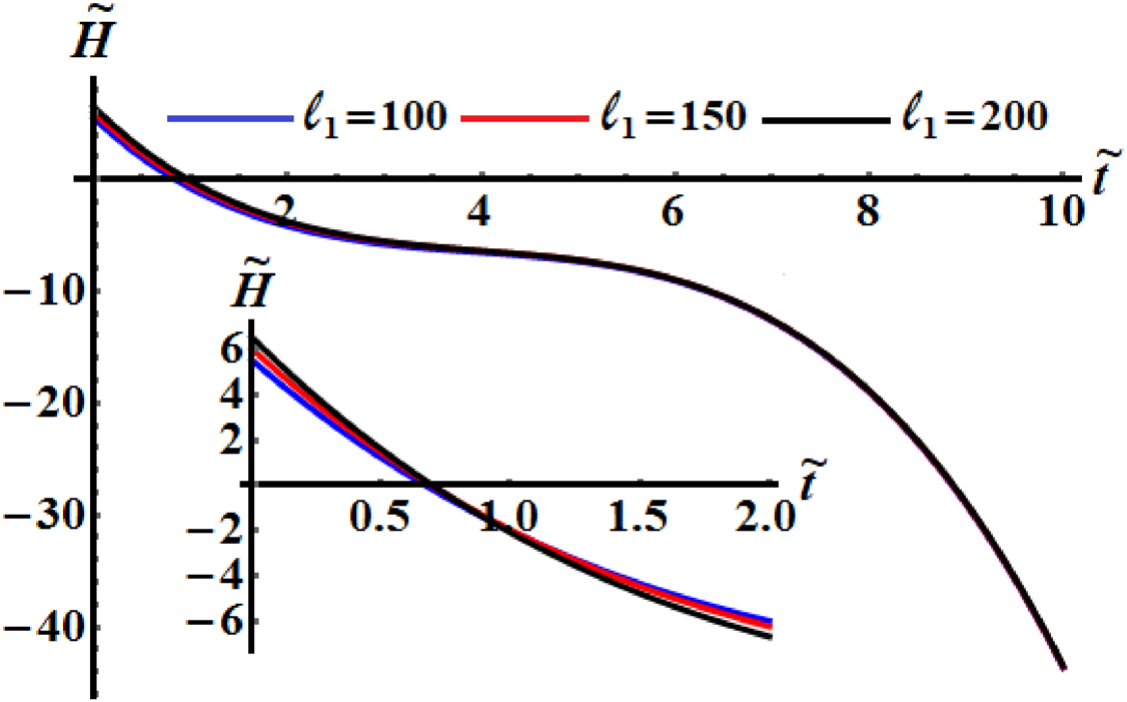
Shows the change of the kinetic energy $\tilde{H}$ over time when $(\ell_{2} ,\ell_{3} ) = (150,50)\;{\text{kg}}\;{\text{m}}^{2} \;{\text{s}}^{ - 1}$ at $\ell_{1} ( = 100,\,150,200)\;{\text{kg}}\;{\text{m}}^{2} \;{\text{s}}^{ - 1} .$

**Figure 6:**
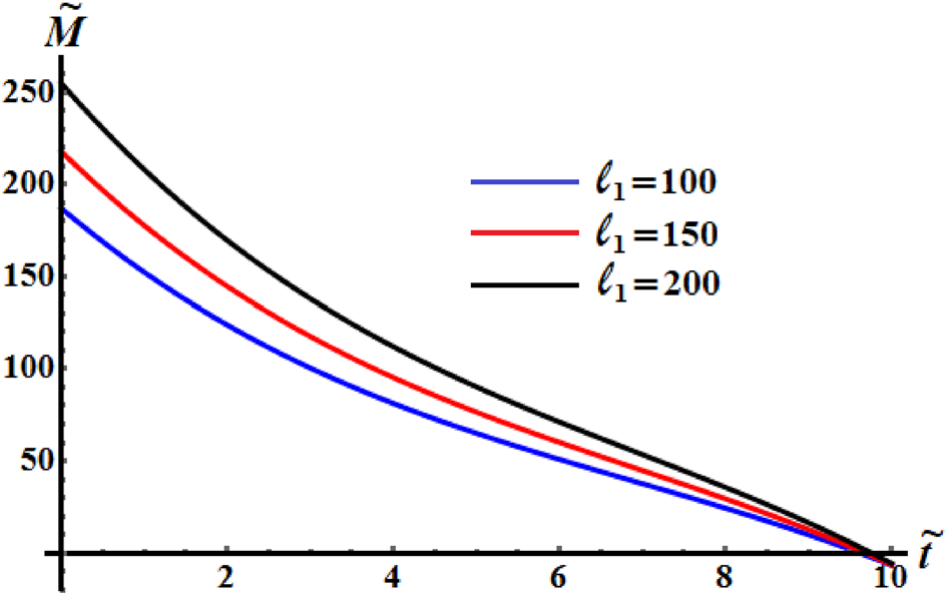
Displays the change of $\tilde{M}$ over time when $(\ell_{2} ,\ell_{3} ) = (150,50)\;{\text{kg}}\;{\text{m}}^{2} \;{\text{s}}^{ - 1}$ at $\ell_{1} ( = 100,\,150,200)\;{\text{kg}}\;{\text{m}}^{2} \;{\text{s}}^{ - 1} .$

**Figure 7:**
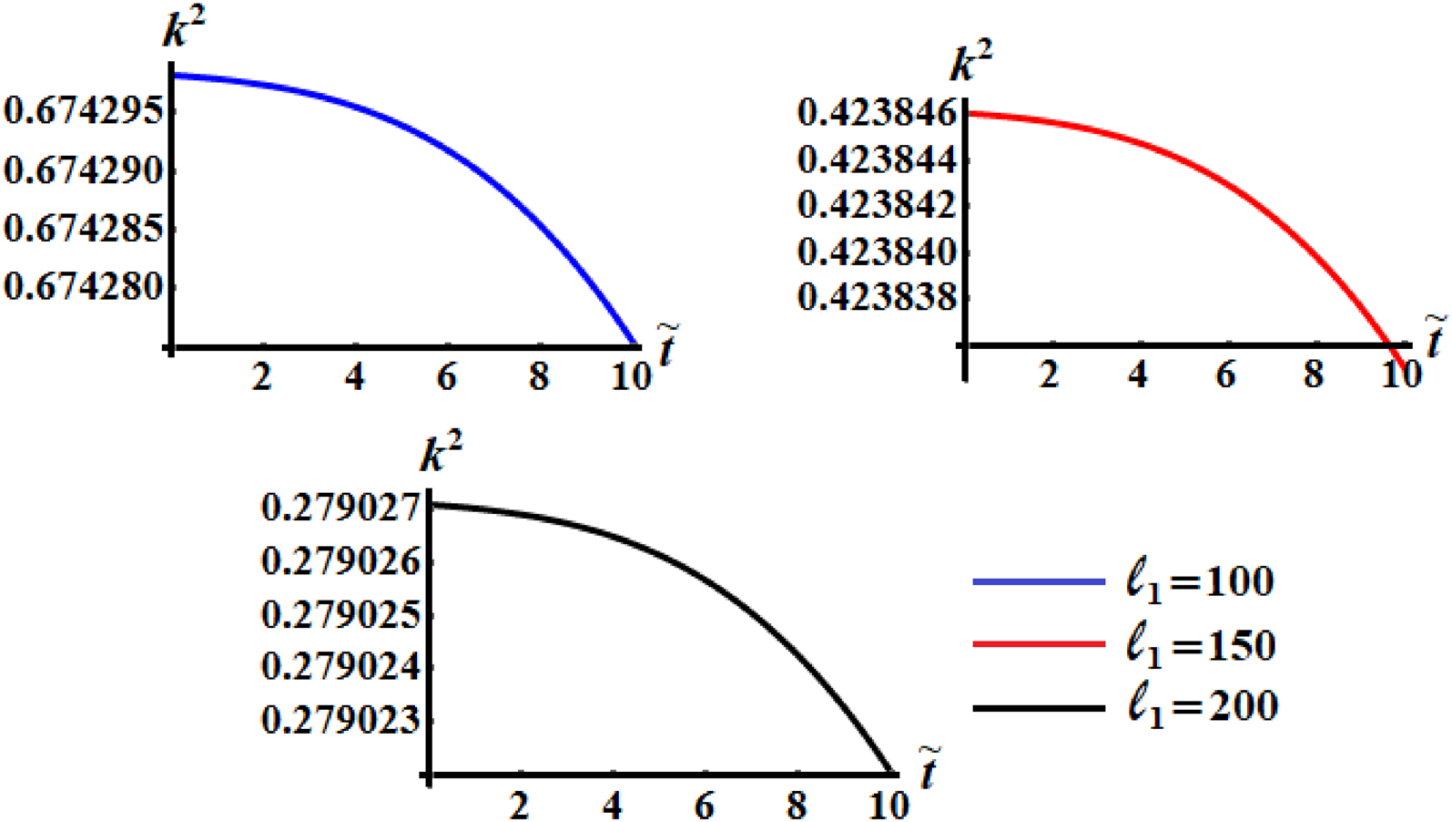
Demonstrates the variation of $k^{2}$ over time when $(\ell_{2} ,\ell_{3} ) = (150,50)\;{\text{kg}}\;{\text{m}}^{2} \;{\text{s}}^{ - 1}$ at $\ell_{1} ( = 100,150,200)\;{\text{kg}}\;{\text{m}}^{2} \;{\text{s}}^{ - 1} .$

It is worthy to mention that the numerical analysis was performed on the identical mass geometry and resistive medium of the RB. The influence of the components of the GM on the kinetic energy $$\tilde{H}$$, the angular moment $$\tilde{M}$$, and the square module $$k^{2}$$ of the RB motion is examined. Therefore, two of them will be constant values while the other component varies. Figures 5, 6, and 7 are calculated at $$\ell_{2} = 150\;{\text{kg}}\;{\text{m}}^{2} \;{\text{s}}^{ - 1} ,$$ $$\ell_{3} = 50\;{\text{kg}}\;{\text{m}}^{2} \;{\text{s}}^{ - 1} ,$$$$\kappa_{1} = 0.5,$$ $$\kappa_{2} = 0.8$$ since $$b_{1} = 5,\,b_{2} = 8,\,b_{3} = 10$$ and $$\sigma = 1.3$$ when the first projection of the GM has the various values $$\ell_{1} ( = 100,150,200)\;{\text{kg}}\;{\text{m}}^{2} \;{\text{s}}^{ - 1}$$. These figures show the changes in the body’s functions $$\tilde{H},\tilde{M},$$ and $$k^{2}$$ with time in the presence of the applied torque and moment. It is clear that the behavior of these functions during the examined time interval has a decay manner for the investigated case of quasi-optimal slowing motion of the RB. An examination of the curves in Figs. 5 and 6 reveals that the inner curvature of these curves increases with the increase of $$\ell_{1}$$ values. These curvatures can be distinguished with the graphed curves of Fig. 6 than the curves of Fig. 5, which highlights the significance of the GM and the control torque the motion of the body. This means that, the process of deceleration can occur at a variety of different values of $$\ell_{1}$$. On the other hand, the curvature of the time variation of $$k^{2}$$ seems to be outer and decreases as the value of $$\ell_{1}$$ rises, see Fig. 7. The increasing or decreasing of the curve’s curvature, whether concave or convex, is due to the mathematical forms of the represented functions as in the system of Eq. (27).

**Figure 8:**
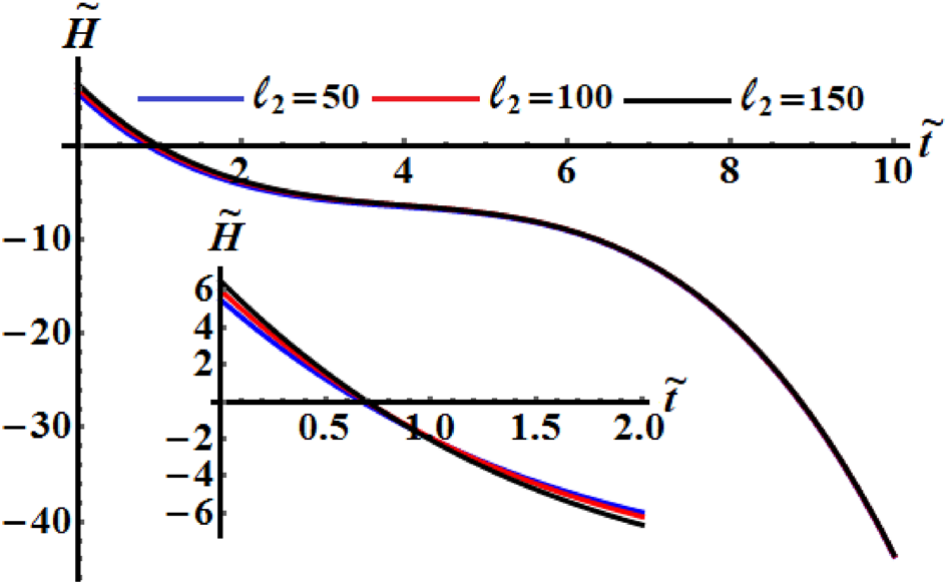
Describes the the kinetic energy $\tilde{H}(\tilde{t})$ when $\ell_{1} = 200\;{\text{kg}}\;{\text{m}}^{2} \;{\text{s}}^{ - 1}$ and $\ell_{3} = 50\;{\text{kg}}\;{\text{m}}^{2} \;{\text{s}}^{ - 1}$ at $\ell_{2} ( = 50,\,100,150)\;{\text{kg}}\;{\text{m}}^{2} \;{\text{s}}^{ - 1} .$

**Figure 9:**
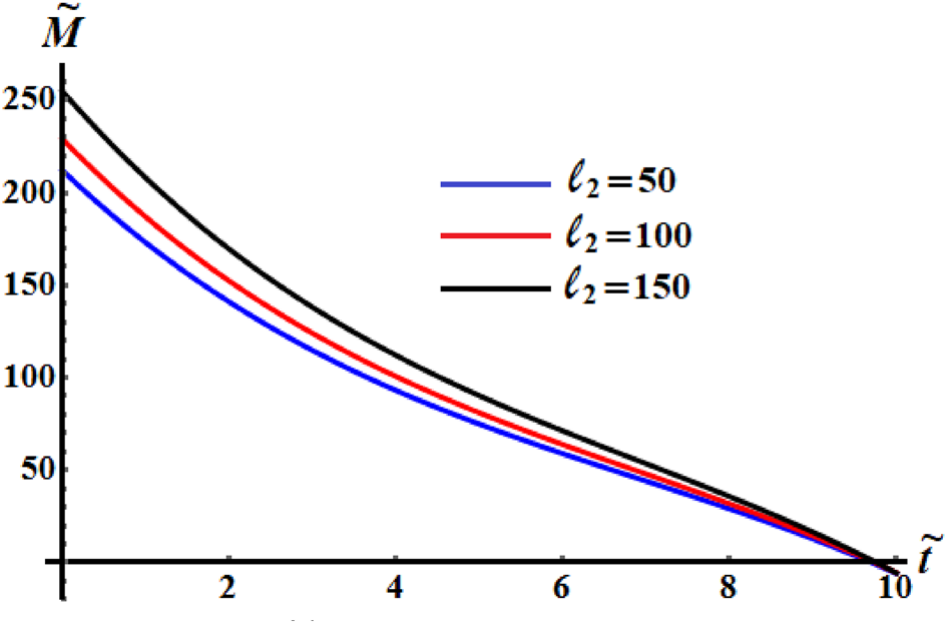
Exhibits the solutions $\tilde{M}(\tilde{t})$ when $\ell_{1} = 200\;{\text{kg}}\;{\text{m}}^{2} \;{\text{s}}^{ - 1}$ and $\ell_{3} = 50\;{\text{kg}}\;{\text{m}}^{2} \;{\text{s}}^{ - 1}$ at $\ell_{2} ( = 50,\,100,150)\;{\text{kg}}\;{\text{m}}^{2} \;{\text{s}}^{ - 1} .$

**Figure 10:**
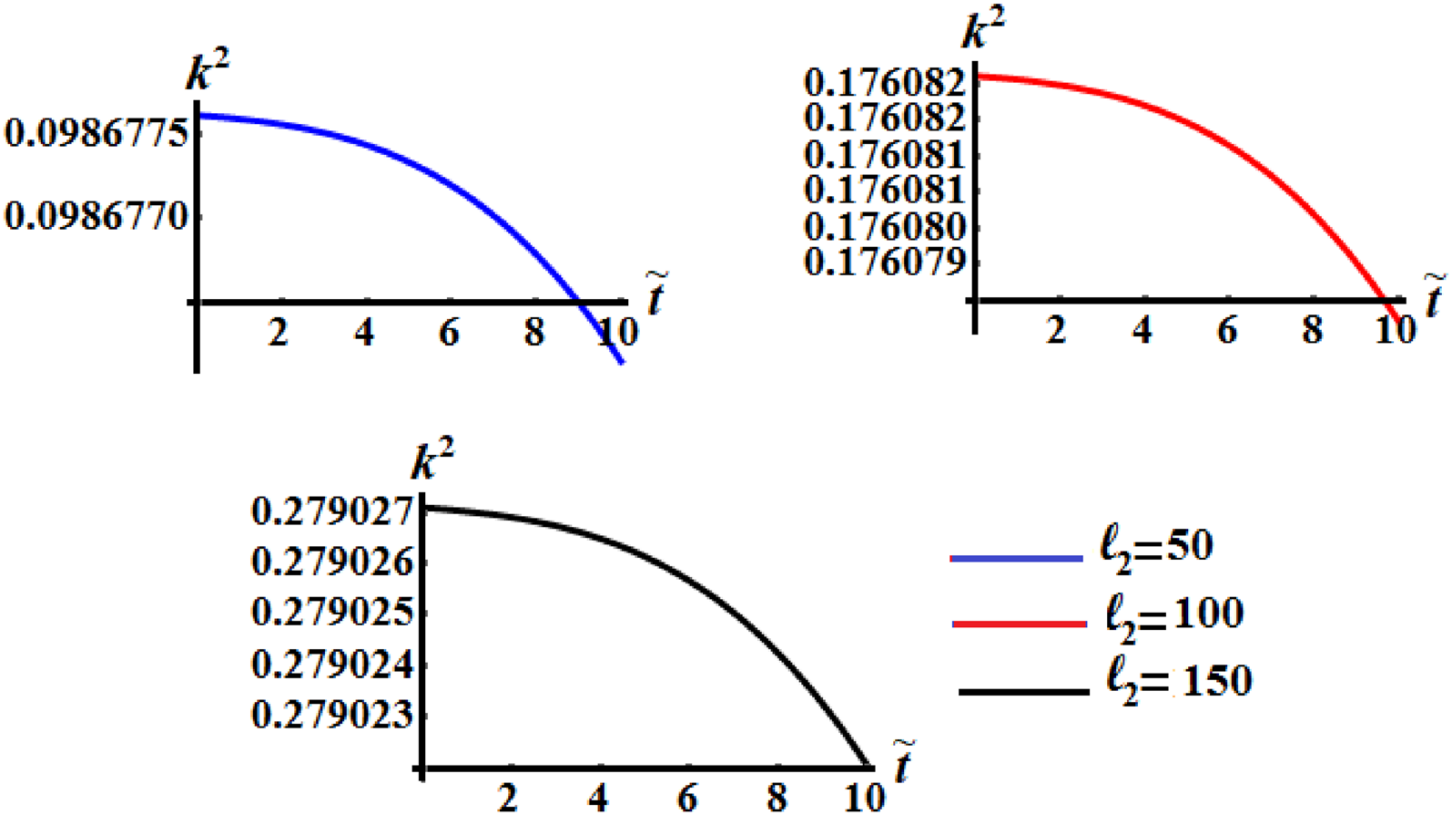
Exhibits the change of $k^{2}$ over time when $\ell_{1} = 200\;{\text{kg}}\;{\text{m}}^{2} \;{\text{s}}^{ - 1}$ and $\ell_{3} = 50\;{\text{kg}}\;{\text{m}}^{2} \;{\text{s}}^{ - 1}$ at $\ell_{2} ( = 50,\,100,150)\;{\text{kg}}\;{\text{m}}^{2} \;{\text{s}}^{ - 1} .$

It is worthwhile to mention that Figs. 8, 9, and 10 are calculated at $$\ell_{1} = 200\;{\text{kg}}\;{\text{m}}^{2} \;{\text{s}}^{ - 1} ,$$ $$\ell_{3} = 50\;{\text{kg}}\;{\text{m}}^{2} \;{\text{s}}^{ - 1} ,$$$$\sigma = 1.3,$$$$\kappa_{1} = 0.5,$$ and $$\kappa_{2} = 0.8$$ where $$b_{1} = 5,\,b_{2} = 8,\,b_{3} = 10$$ when $$\ell_{2}$$ has the different selected values 50, 100 and 150 kg m^2^ s^−1^. It is noted that curvature of the functions of kinetic energy $$\tilde{H}$$, angular momentum $$\tilde{M}$$, and the square module $$k^{2}$$ curves increase with the increase of $$\ell_{2}$$ that mean the time of the optimal deceleration of RB is increasing. The function $$\tilde{H}$$ is uniformly goes way down in the quasi-optimal duration of time $$\tilde{t}$$ in all numerically examined circumstances when $$\ell_{2}$$ has the above considered values, as seen in Figs. 8 and 9. In other words, the slowing process can occur at a range of various values of $$\ell_{2}$$. A closer look at the curves of Fig. 10 explore that the larger of the control torque yields faster deceleration of the RB, and the plots become more complex, with concave and convex portions plainly visible. Moreover, the convexity of the curvature increases with the increase of the second projection of the GM on the corresponding principal axis.

**Figure 11:**
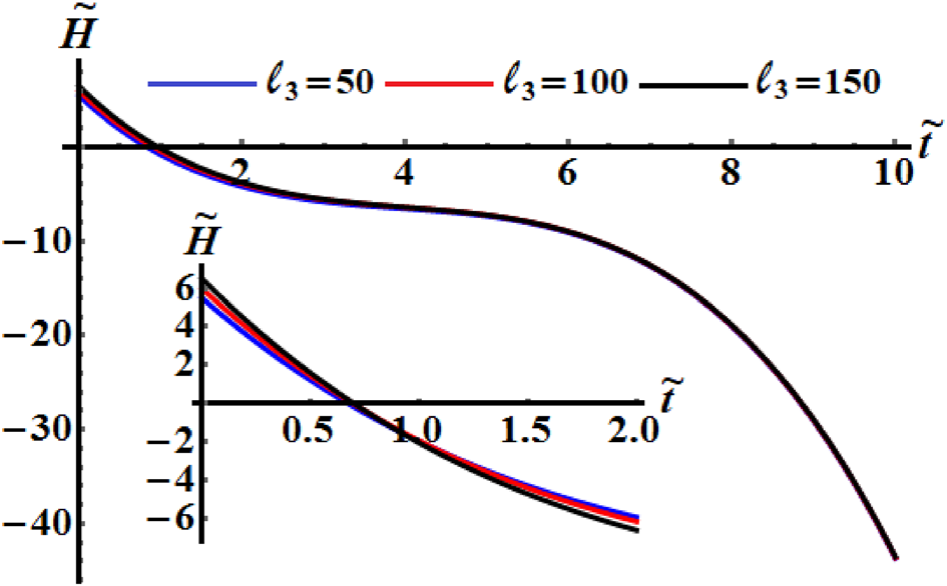
Illustrates $\tilde{H}(\tilde{t})$ when $(\ell_{1} ,\ell_{2} ) = (200,150)\;{\text{kg}}\;{\text{m}}^{2} \;{\text{s}}^{ - 1}$ at $\ell_{3} ( = 50,\,100,150)\;{\text{kg}}\;{\text{m}}^{2} \;{\text{s}}^{ - 1} .$

**Figure 12:**
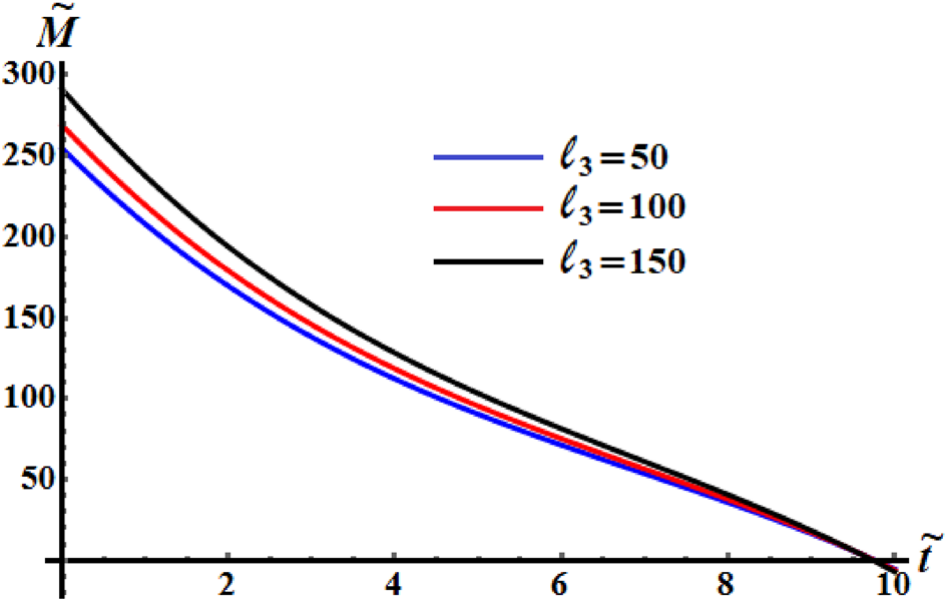
Demonstrates $\tilde{M}(\tilde{t})$ when $(\ell_{1} ,\ell_{2} ) = (200,150)\;{\text{kg}}\;{\text{m}}^{2} \;{\text{s}}^{ - 1}$ at $\ell_{3} ( = 50,\,100,150)\;{\text{kg}}\;{\text{m}}^{2} \;{\text{s}}^{ - 1} .$

**Figure 13:**
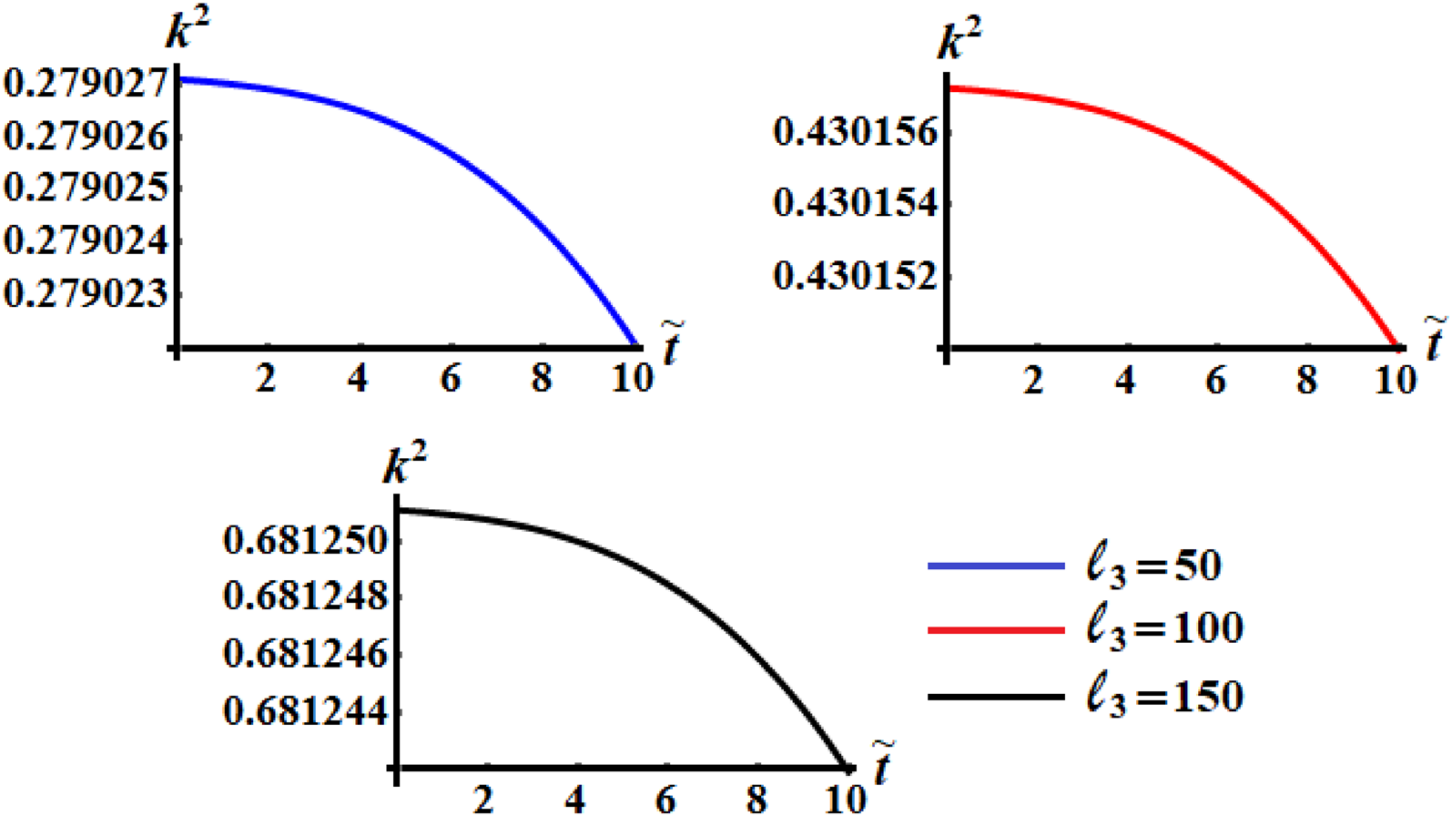
Describes the time history of $k^{2}$ when $(\ell_{1} ,\ell_{2} ) = (200,150)\;{\text{kg}}\;{\text{m}}^{2} \;{\text{s}}^{ - 1}$ a $\ell_{3} ( = 50,\,100,150)\;{\text{kg}}\;{\text{m}}^{2} \;{\text{s}}^{ - 1} .$

Figures 11, 12, and 13 exhibit the time dependent variation of the kinetic energy $\tilde{H}$, angular momentum $\tilde{M}$, and the square module $k^{2}$ at $(\ell_{1} ,\ell_{2} ) = (200,150){\kern 1pt} \;{\text{kg}}\;{\text{m}}^{2} \;{\text{s}}^{ - 1}$ when $\ell_{3} ( = 50,100,150)\;{\text{kg}}\;{\text{m}}^{2} \;{\text{s}}^{ - 1}$ in addition to the above considered values of the parameters. It is clear to see from the included curves in Fig. 10 that the increase of $\ell_{3}$ values yields an increase of the curvature of $\tilde{H}$ which reveals the good influence of these components of the GM as well as the other two components. This function decreases to its zero during the time interval $(0.8,1]$ and then it behaves a monotonically decreasing when $\tilde{t} \in (1,6]$. After that the deceline of the curves becomes sharp during the interval $(6,10]$. The fast slowing of the curves describing the variation of the function $\tilde{M}$ with time is observed when $\ell_{3}$ increases as drawn in Fig. 12. The intial values of $\tilde{M}$ increase with the increasing of $\ell_{3}$ values and the deceleration become faster, while the curvature of the convex curves increases with the decrease of these values. The curvature of the curves included in Fig. 13 decreases with the increase of $\ell_{3}$ values.

It is important to note that, when the gyrostatic moment vanishes, i.e., $\underline{\ell } \equiv \underline{0}$, it violates the elliptic condition, and we can't draw any plots to make a comparison with the results in^28^ for the used initial values. In other words, in the presence of $\ell_{1} ,\ell_{2} ,$ and $\ell_{3} ,$ the condition $0 \le k^{2} \le 1$ holds, while it isn’t satisfied in the absence of the gyrostatic moment as in^28^ and the solutions will be imaginary. This confirms the importance of the effect of the gyrostat moment on the problem under study.

**Figure 14:**
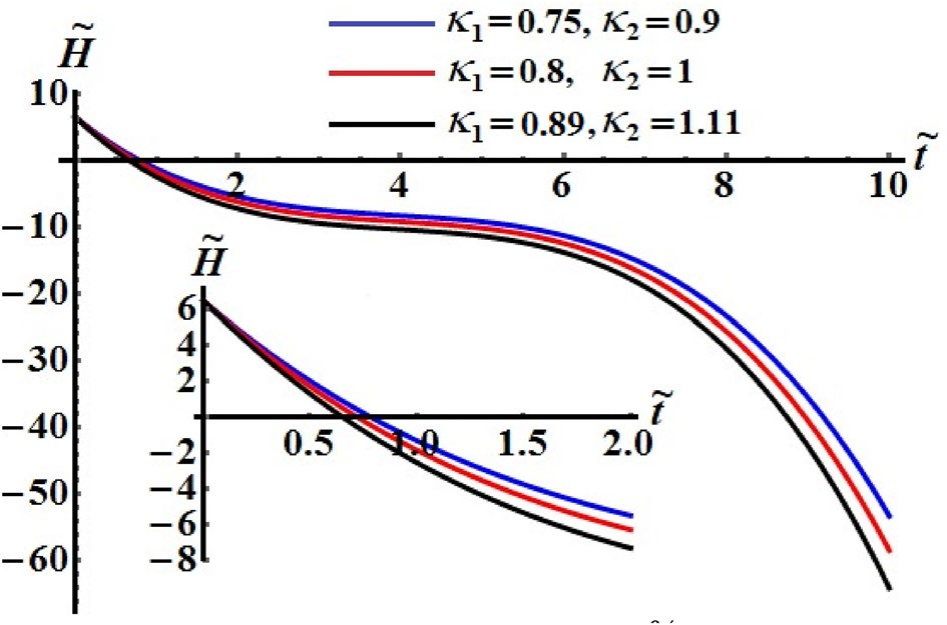
Depicts the temporal history of $\tilde{H}$ at $\sigma = 1.3$.

**Figure 15:**
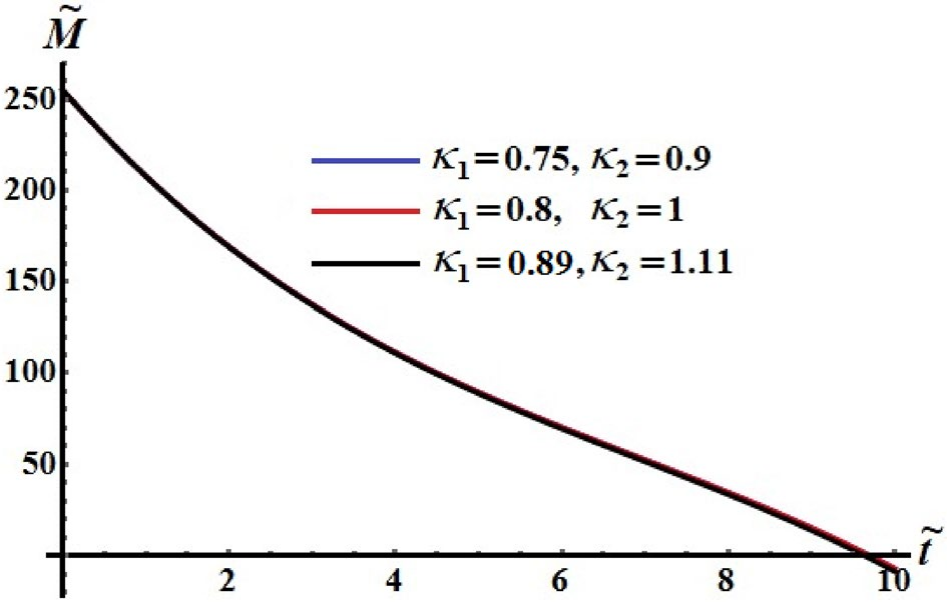
Depicts the temporal history of $\tilde{M}$ at $\sigma = 1.3$.

**Figure 16:**
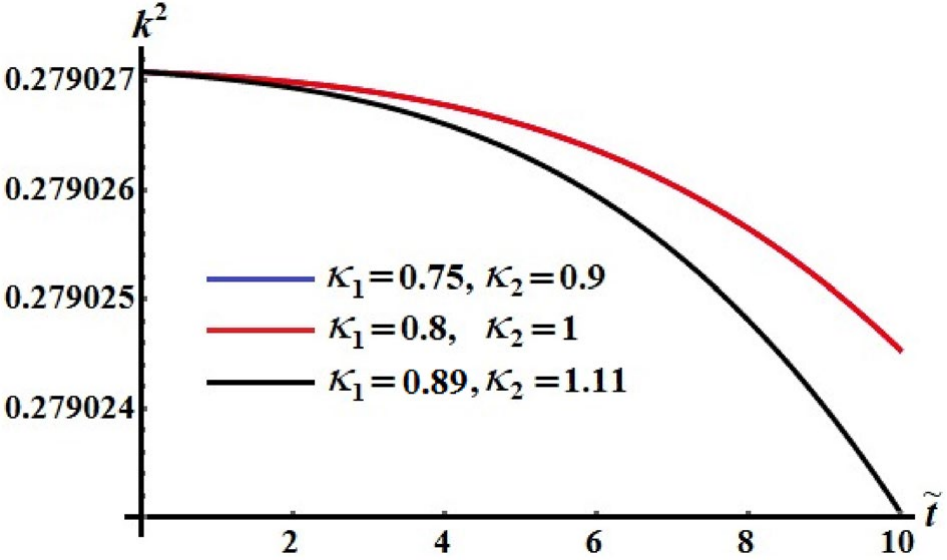
Portrays the temporal history of $k^{2}$ at $\sigma = 1.3$.

The numerical computations for the computed values of the dimensionless components of the control torque $\kappa_{1}$ and $\kappa_{2}$ are performed when the identifing number $\sigma$ has different values. Therefore, Figs. 14, 15, and 16 are drawn at $\sigma = 1.3$ when $\ell_{1} ,\ell_{2} ,$ and $\ell_{3}$ have the values 200, 150, and 50, respectively. Here, the values of $(\kappa_{1} ,\kappa_{2} )$ are $(0.75,0.9),$ $(0.8,1),$ and $(0.89,1.11)$. The corresponding values of these figures for the torques $(b_{1} ,b_{2} ,b_{3} )$ are $(15,18,20)$, $(20,25,25)$, and $(20,25,25)$, respectively. When $\kappa_{j} \,(j = 1,2)$ increase, the curvature of the plotted curves of the kinetic energy function increases, as seen in Fig. 14. Moreover, the first values of them produce an almost linear curve. On the other hand, the curves of the functions of angular momentum coincide with each other to some extent, as graphed in Fig. 15, while the plotted curves of $k^{2}$ are characterized for the values of $\kappa_{1}$ and $\kappa_{2}$ as observed in the curves of Fig. 16.

**Figure 17:**
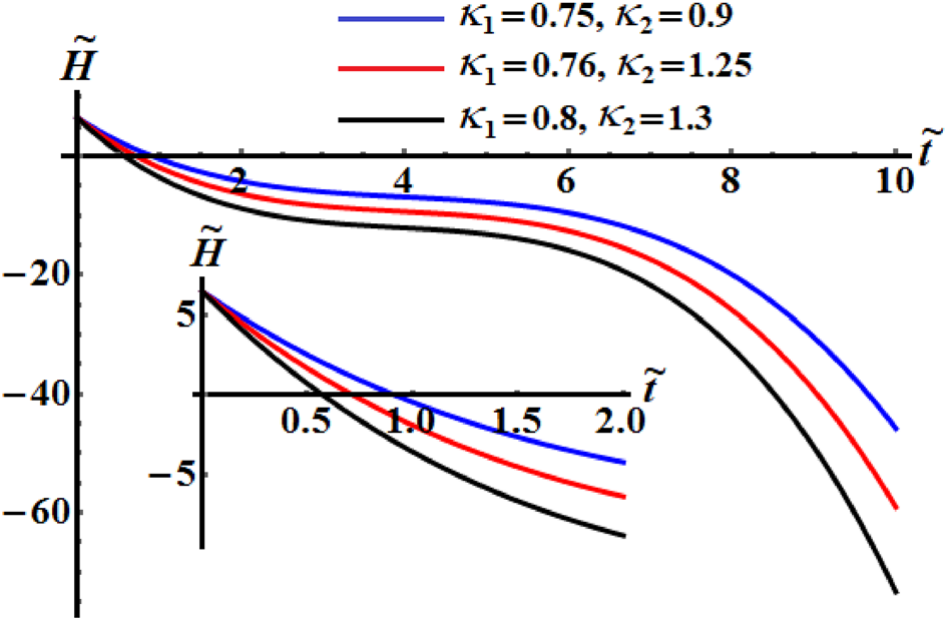
Reveals the variation of $\tilde{H}$ with $\tilde{t}$ at $\sigma = 1.8.$

**Figure 18:**
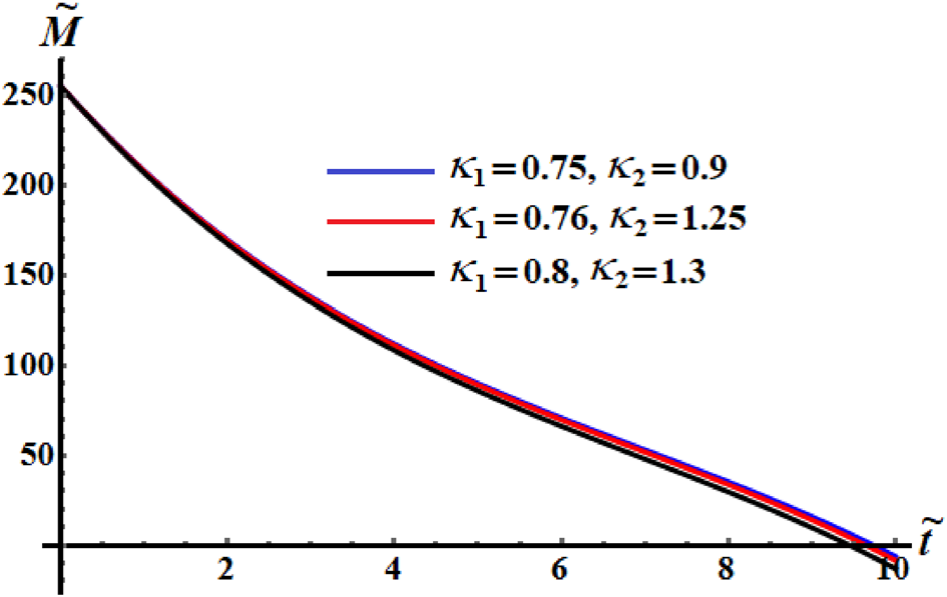
Reveals the variation of $\tilde{M}$ with $\tilde{t}$ at $\sigma = 1.8.$

**Figure 19:**
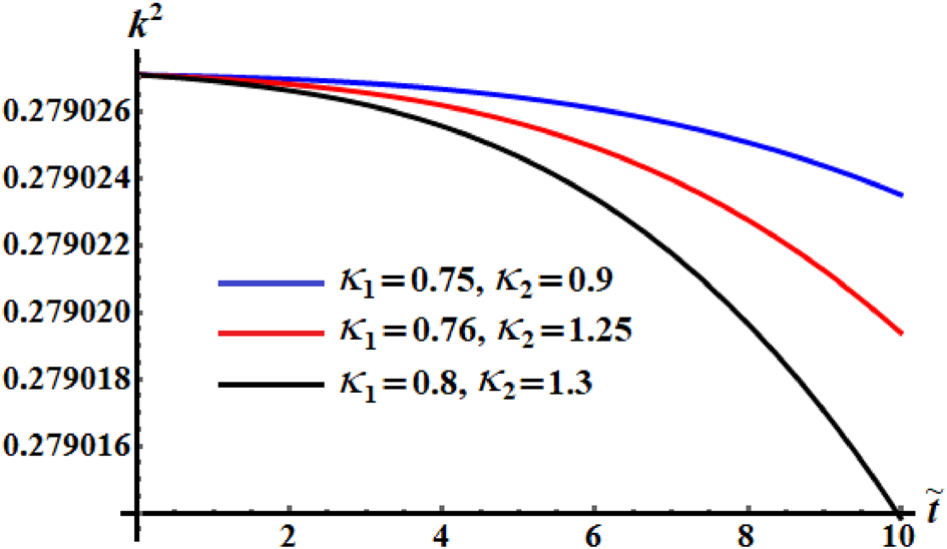
Describes the change of $k^{2}$ over time $\tilde{t}$ at $\sigma = 1.8.$

Curves of Figs. 17, 18, and 19 are calculated at $(\kappa_{1} ,\kappa_{2} ) = (0.89,1.3)$, $(\kappa_{1} ,\kappa_{2} ) = (0.76,1.25)$ and $(\kappa_{1} ,\kappa_{2} ) = (0.89,1.3)$, respectively. The corresponding values of these figures for the torques are $(b_{1} ,b_{2} ,b_{3} ) = (15,18,20),$ $(b_{1} ,b_{2} ,b_{3} ) = (15.2,25,20),$ and $(b_{1} ,b_{2} ,b_{3} ) = (16,25,18),$ respectively. Moreover, $\sigma = 1.8$ and the same considered values of $\ell_{1} ,\ell_{2} ,$ and $\ell_{3}$ in the previous three figures, are taken into account. These figures explore the temporal histories of the functions $\tilde{H},$ $\tilde{M},$ and $k^{2}$. When Figs. 14, 15, 16 are compared with Figs. 17, 18, 19, we can see the curves of Figs. 17, 18, 19 are more distinguished than the included curves of Figs. 14, 15, 16. This distinction between these curves is due to the change of the identifying number $\sigma$ which reveals the significance of this number.

**Figure 20:**
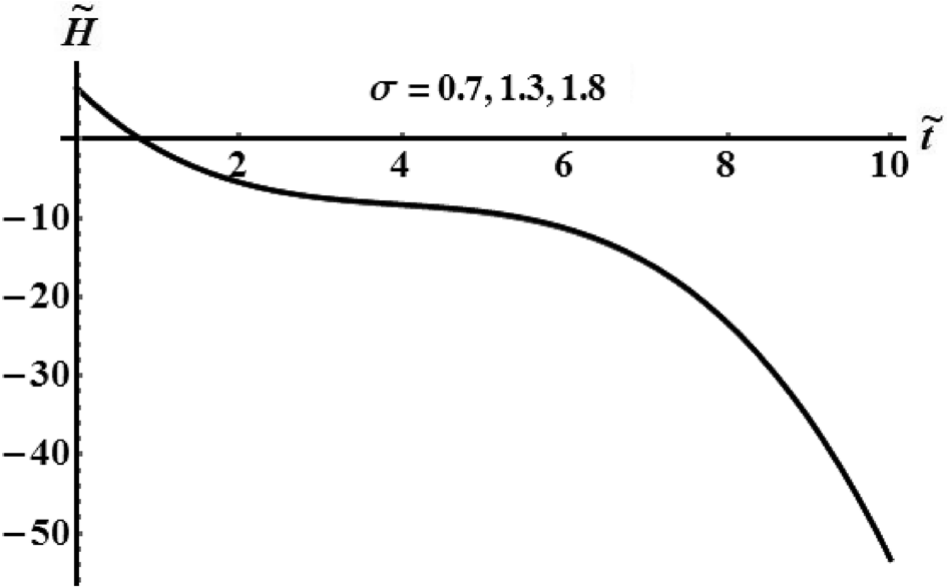
Shows the evolve of $\tilde{H}$ over time $\tilde{t}$ at $\sigma \,( = 0.7,\,1.3,\;1.8).$

**Figure 21:**
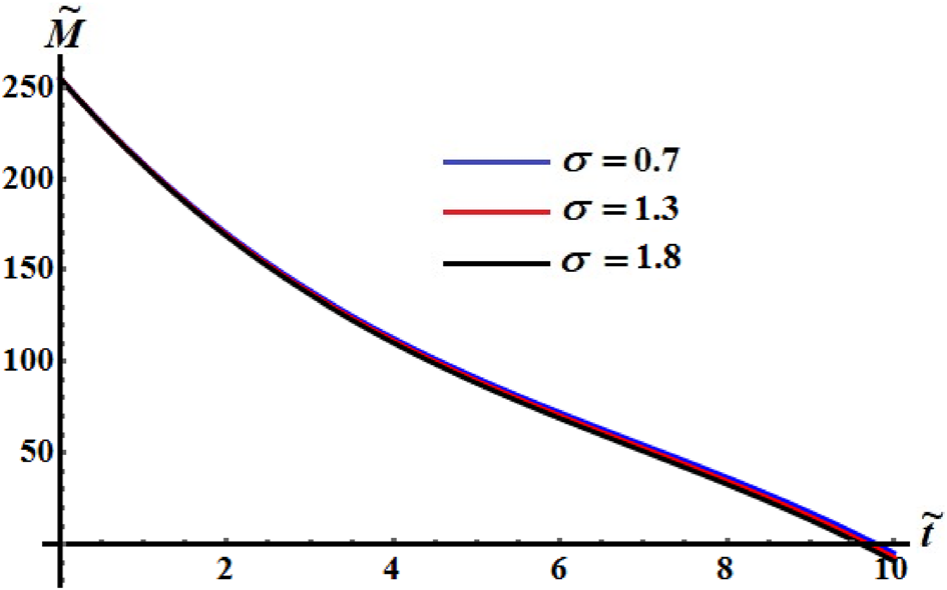
Shows the evolve of $\tilde{M}$ over time $\tilde{t}$ at $\sigma \,( = 0.7,\,1.3,\;1.8).$

**Figure 22:**
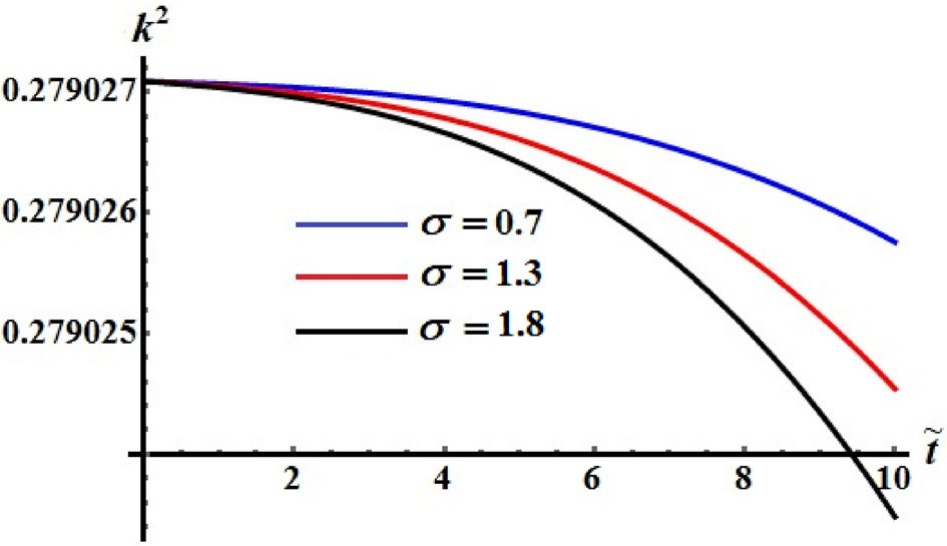
Portrays the evolve of $k^{2}$ over time $\tilde{t}$ at $\sigma \,( = 0.7,\,1.3,\;1.8).$

The influence of various values of $\sigma$ number is drawn in Figs. 20, 21, and 22 for the functions $\tilde{H},$ $\tilde{M},$ and $k^{2}$, respectively. These curves show the numerical computations of the system (26), in which there is no variation of the kinetic energy curves with the various values of $\sigma$, see Fig. 20. Whereas the functions of angular momentum and square module are varied over the used time intervals, as seen in Figs. 21 and 22. The time of deceleration decreases with the increase of the values of $\sigma$.

## Impact of a tiny perturbation

This section presents a sensitivity analysis to study the impact of a tiny perturbation and to examine the robustness of the model. In^47^, the authors investigated the optimal slowing of an asymmetric body's rotations in a resistant medium

30 $$b_{1} = b_{2} = b_{3} = b.$$

In that study, the time change of the angular momentum vector's magnitude and the kinetic energy of RB are found analytically. The solution of system (27) in the presence of the circumistantes (30) yields results that are in convergence with the obtained ones in^47^. Now, let's examine how these functions behave when the control torque coefficients are varied by a tiny variation. Based on Eq. (20), the dimensionless coefficients $\kappa_{j} \,(j = 1,2)$ can be introduced and the third one $\kappa_{3}$ can be used as unity.

Therefore, small increments $\left| {\mu_{i} } \right| < < 1$ are considered as follows

31 $$\kappa_{j} = 1 + \mu_{j}; \,\,\,j = 1,2.$$

**Figure 23:**
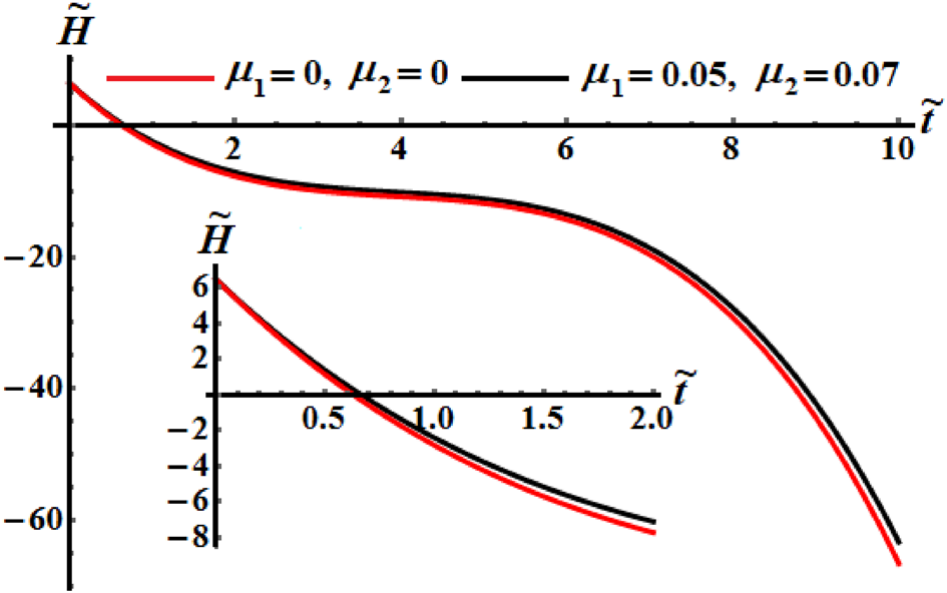
Portrays the evolve of $\tilde{H}$ over time $\tilde{t}$ at $\mu_{j} = 0\,\,(j = 1,2),$ and $\mu_{1} = 0.05,\mu_{2} = 0.07.$

**Figure 24:**
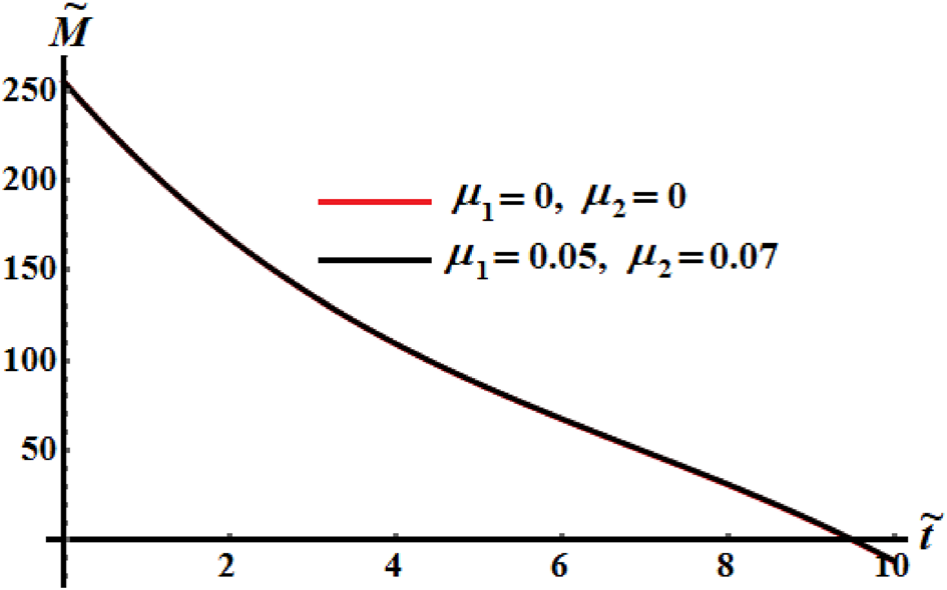
Portrays $\tilde{M}(\tilde{t})$ at $\mu_{j} = 0\,\,(j = 1,2),$ and $\mu_{1} = 0.05,\mu_{2} = 0.07.$

**Figure 25:**
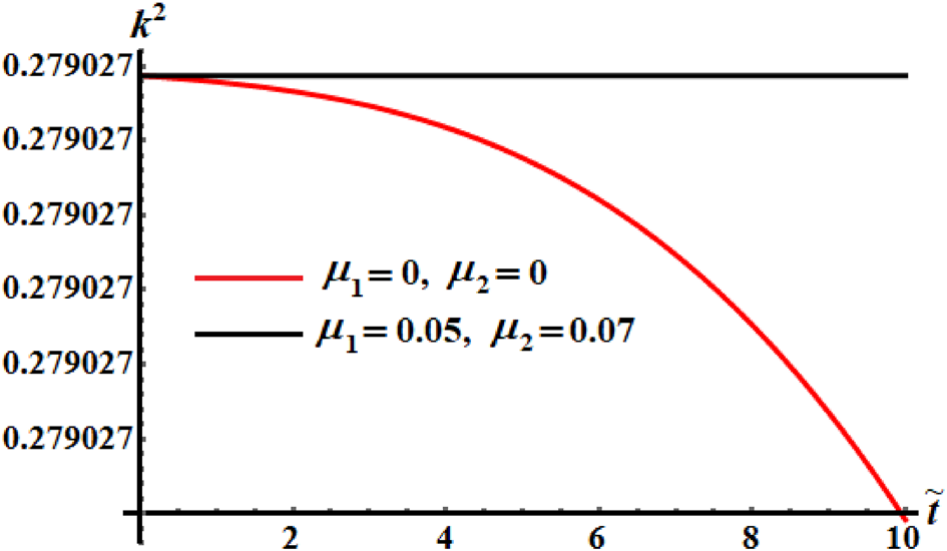
Provides the temporal history of $k^{2}$ at $\mu_{j} = 0\,\,(j = 1,2),$ and $\mu_{1} = 0.05,\mu_{2} = 0.07.$

Curves of Figs. 23, 24, and 25 are plotted for the comparison between the numerical investigation of the coefficients of the control torques at $\mu_{j} = 0$, and the perturbed ones at $\mu_{1} = 0.05$ and $\mu_{2} = 0.07$. A small deviation between them is noticed. We may conclude from these plots that any tiny changes in one of the coefficients produces a little increase in the deceleration function of the RB.

## Concluding remarks

Analytically and numerically, a time quasi-optimal slowing problem for the rotation of a dynamically asymmetric RB in a resistive medium under the influence of a GM and a tiny control torque is examined. The control optimal decelerating law for the RB rotation is achieved, in which the study of quasi-stationary trajectories is carried out. The governing mechanism system of motion is obtained in its dimensionless form. The obtained novel results are depicted to determine the GM’s favourable impact on the functions of the angular momentum and the kinetic energy, as well as the square module. The terms of “optimal” and “semi-optimal” slowing are different when different values of the controlled torque dimension are selected. A case of tiny perturbation is studied to reveal the action of the control torques on the body’s motion and it is compared with the unperturbed one. The achieved results are graphed for various values of the GM, identifying number, and perturbed parameter. These results generalize those which were obtained in^28^ for the zero value of the GM. These out comes are significant because they can be used to regulate aviation and submarine systems.

## Data Availability

The datasets used and/or analysed during the current study available from the corresponding author on reasonable request.

## References

[1] Leimanis E (1965). The General Problem of the Motion of Coupled Rigid Bodies About a Fixed Point.

[2] Yehia HM (1986). On the integrability of certain problems in particle and rigid body dynamics. J. Theoret. Appl. Mech..

[3] Amer TS, Amer WS (2018). The substantial condition for the fourth first integral of the rigid body problem. Math. Mech. Solids.

[4] Elmandouh AA (2018). New integrable problems in a rigid body dynamics with cubic integral in velocities. Results Phys..

[5] Arkhangel’skii AI (1963). On the motion about a fixed point of a fast spinning heavy solid. J. Appl. Math. Mech..

[6] Ismail AI, Amer TS (2002). The fast spinning motion of a rigid body in the presence of a gyrostatic momentum. Acta Mech..

[7] Amer WS (2021). Modelling and analyzing the rotatory motion of a symmetric gyrostat subjected to a Newtonian and magnetic fields. Results Phys..

[8] Elfimov VS (1978). Existence of periodic solutions of equations of motion of a solid body similar to the lagrange gyroscope. J. Appl. Math. Mech..

[9] Amer TS (2017). On the dynamical motion of a gyro in the presence of external forces. Adv. Mech. Eng..

[10] Amer TS (2008). On the motion of a gyrostat similar to Lagrange's gyroscope under the influence of a gyrostatic moment vector. Nonlinear Dyn..

[11] Amer TS, Galal AA, Abady IM, El-Kafly HF (2021). The dynamical motion of a gyrostat for the irrational frequency case. Appl. Math. Model..

[12] Amer TS, Abady IM (2018). On the motion of a gyro in the presence of a Newtonian force field and applied moments. Math. Mech. Solids.

[13] Galal AA, Amer TS, El-Kafly H, Amer WS (2020). The asymptotic solutions of the governing system of a charged symmetric body under the influence of external torques. Results Phys..

[14] El-Sabaa FM, Amer TS, Sallam AA, Abady IM (2022). Modeling and analysis of the nonlinear rotatory motion of an electromagnetic gyrostat. Alex. Eng. J..

[15] Amer TS, Abady IM (2017). On the application of KBM method for the 3-D motion of asymmetric rigid body. Nonlinear Dyn..

[16] Amer TS, El-Kafly HF, Galal AA (2021). The 3D motion of a charged solid body using the asymptotic technique of KBM. Alex. Eng. J..

[17] Amer TS, Farag AM, Amer WS (2020). The dynamical motion of a rigid body for the case of ellipsoid inertia close to ellipsoid of rotation. Mech. Res. Commu..

[18] El-Sabaa FM (1985). A new class of periodic solutions in the Kovaleveskaya case of a rigid body in rotation about a fixed point. Celestial Mech..

[19] El-Sabaa MF (1993). About the periodic solutions of a rigid body in a central Newtonian field. Celest. Mech. Dyn. Astron..

[20] Amer TS, Amer WS (2018). The rotational motion of a symmetric rigid body similar to Kovalevskaya’s case. Iran. J. Sci. Technol. Trans. Sci..

[21] El-Sabaa FM, Amer TS, Gad HM, Bek MA (2021). Existence of periodic solutions and their stability for a sextic galactic potential function. Astrophys. Space Sci..

[22] Akulenko LD, Leshchenko DD (1995). Optimal deceleration of rotation of a solid with internal degrees of freedom. Comp. Syst. Sci. No..

[23] Akulenko LD (1987). Asymptotic Methods of Optimal Control.

[24] Akulenko LD, Leshchenko DD, Rachinskaya AL (2010). Optimal deceleration of rotation of a dynamically symmetric body with a cavity filled with viscous liquid in a resistive medium. J. Comput. Syst. Sci. Int..

[25] Akulenko LD, Zinkevich YS, Leshchenko DD (2011). Optimal rotation deceleration of a dynamically asymmetric body in a resistant medium. J. Comput. Syst. Sci. Int..

[26] Akulenko LD, Leshchenko DD, Rachinskaya AL (2012). Optimal deceleration of rotations of an asymmetric body with a cavity filled with viscous fluid in a resistive medium. J. Comput. Syst. Sci. Int..

[27] Akulenko LD, Zinkevich YS, Leshchenko DD, Rachinskaya AL (2011). Optimal rotation deceleration of a dynamically symmetric body with movable mass in a resistant medium. J. Comput. Syst. Sci. Int..

[28] Akulenko LD, Leshchenko DD, Rachinskaya AL (2014). Quasi-optimal deceleration of rotations of an asymmetric body in resistive medium. J. Comput. Syst. Sci. Int..

[29] Akulenko LD, Kozachenko TA, Leshchenko DD (2018). Quasi-optimal braking of rotations of a body with a moving mass coupled to it through a quadratic friction damper in a resisting medium. J. Comput. Syst. Sci. Int..

[30] Akulenko LD, Kozachenko TA, Leshchenko DD (2019). Time quasi-optimal deceleration of rotations of a gyrostat with a moving mass in a resistive medium. J. Comput. Syst. Sci. Int..

[31] Akulenko LD, Leshchenko DD, Chernous’ko FL (1982). Fast motion of a heavy rigit body about a fixed point in a resistive medium. Mech. Solids.

[32] Akulenko LD, Leshchenko DD, Rachinskaya AL (2008). Evolution of the satellite fast rotation due to the gravitational torque in a dragging medium. Mech. Solids.

[33] Koshlyakov VN (1985). Problems in Dynamics of Solid Bodies and in Applied Gyroscope Theory: Analytical Methods.

[34] Inarrea M, Lanchares V (2006). Chaotic pitch motion of an asymmetric non-rigid spacecraft with viscous drag in circular orbit. Int. J. Non-Linear Mech..

[35] Akulenko LD (1994). Problems and Methods of Optimal Control.

[36] Nayfeh AH (2004). Perturbations Methods.

[37] Malkin IG (1959). Some Problems in the Theory of Nonlinear Oscillations (AEC-tr-3766).

[38] Leshchenko DD (1996). Time-optimal damping of rotations of a rigid body with internal degrees of freedom with respect to speed. J. Comput. Syst. Sci. Int..

[39] Zinkevich YS (2016). Quasi-optimal deceleration of rotational motion of a dynamically symmetric rigid body in a resistive medium. Mech. Solids.

[40] Akulenko LD, Leshchenko DD, Shchetinina YS (2017). Quasi-optimal deceleration of rotations of a rigid body with a moving mass in resistive medium. J. Comput. Syst. Sci. Int..

[41] Rachinskaya AL, Rumyantseva EA (2018). Optimal deceleration of a rotating asymmetrical body in a resistive medium. Int. Appl. Mech..

[42] Pukdeboon C (2011). A review of fundamentals of Lyapunov theory. J. Appl. Sci..

[43] Smol’nikov BA (1967). Generalization of Euler case of motion of a solid. J. Appl. Math. Mech..

[44] Chernousko FL, Akulenko LD, Sokolov BN (1980). Control of Oscillations.

[45] Landau LD, Lifshitz EM (1993). Mechanics.

[46] Gradshtein IS, Ryzhik IM (2014). Tables of Integrals, Series, and Products.

[47] El-Sabaa FM, Amer TS, Sallam AA, Abady IM (2022). Modeling of the optimal deceleration for the rotatory motion of asymmetric rigid body. Math. Comput. Simul..

